# A dynamical systems analysis of criminal behavior using national longitudinal survey of youth data

**DOI:** 10.1371/journal.pone.0324014

**Published:** 2025-08-08

**Authors:** David McMillon, Jeffrey Morenoff, Carl Simon, Erin Lane

**Affiliations:** 1 Department of Economics, Emory University, Atlanta, Georgia, United States of America; 2 Stone Center for Research on Wealth Inequality, Chicago, Illinois, United States of America; 3 Federal Reserve Bank of Atlanta, Atlanta, Georgia, United States of America; 4 Gerald R. Ford School of Public Policy, University of Michigan, Ann Arbor, Michigan, United States of America; 5 Department of Sociology, University of Michigan, Ann Arbor, Michigan, United States of America; 6 Institute for Social Research, University of Michigan, Ann Arbor, Michigan, United States of America; 7 Center for the Study of Complex Systems, University of Michigan, Ann Arbor, Michigan, United States of America; 8 Department of Mathematics, University of Michigan, Ann Arbor, Michigan, United States of America; 9 Department of Economics, University of Michigan, Ann Arbor, Michigan, United States of America; 10 ApplEcon, LLC., Ann Arbor, Michigan, United States of America; SUNY Downstate Health Sciences University, UNITED STATES OF AMERICA

## Abstract

Building on previous work on the spread and sustenance of crime, we construct and analyze a dynamical systems model of criminal involvement, arrest, desistance, and rehabilitation to be estimated empirically using interviews in the National Longitudinal Survey of Youth. We examine how marginal increases in flows between states interact to decrease or increase the long-run level of crime, and whether this varies by subgroup. We study how observed racial disparities along certain pathways interact to generate macro-level disparities in criminal involvement as measured by arrest and self-report. Finally, we discuss the implications of the model for a broader policy debate on crime control and for competing explanations of the Black-White gap in criminal involvement. We find, among other conclusions, that marginal independent increases in first-time arrest rates (but not arrest rates for repeat offenders) increase long-run crime for all subgroups; that long-run crime levels for Black men are most sensitive to initial flows into crime and arrest and to rehabilitation; and that among people with no arrest history, Black women are significantly more likely than other subgroups to desist the following year.

## Introduction

Understanding the dynamics of criminal involvement, arrest, desistance, and rehabilitation requires insight into not only the individual drivers of each process, but also the systemic effects and unintended consequences that arise from their interactions. Crime is not a static phenomenon but a dynamic process shaped by interactions between personal circumstances, institutional responses, and broader societal structures. Prior research has established that disparities in criminal justice outcomes—particularly along racial lines—are influenced by variations in exposure to risk factors, differential treatment within the justice system, and uneven access to rehabilitative resources. However, many existing models fail to integrate these elements into a cohesive dynamical systems framework capable of capturing the long-term consequences of marginal changes in key transitions, such as entry into crime, arrest, and rehabilitation.

This paper builds on previous systems-based approaches to crime modeling by constructing and analyzing a dynamical systems model of criminal involvement, arrest, desistance, and rehabilitation. Using empirical estimates derived from the 1997 National Longitudinal Survey of Youth (NLSY97), we examine how small shifts in transition probabilities between different states of criminal involvement affect long-run crime levels. A central focus of this study is the extent to which racial disparities in specific pathways—such as first-time arrests, recidivism, and rehabilitation—contribute to long-run racial gaps in overall criminal involvement.

Our analysis provides several novel insights with direct implications for policy and theory. We find that increases in first-time arrest rates—though often framed as a deterrent—can paradoxically lead to higher long-term crime rates, while increased arrests of those previously arrested has very little effect on the overall crime rate. This is partly due to the already high annual rates of disengagement from crime among people with no arrest history and relatively high rates of recidivism after arrest. We find that Black women are especially likely to follow a pattern of refraining from criminal offending without arrest. Furthermore, our results indicate that the long run crime rate among all subgroups is sensitive to changes in the rate at which people initially start offending and that Black men’s long-run crime trajectories are particularly sensitive to access to rehabilitation opportunities. These findings challenge conventional narratives about crime control and illustrate how the interdependence of criminological processes can lead to nuanced and counterintuitive consequences for policy interventions.

We offer a systems-based perspective on the persistent racial disparities in criminal involvement, situating our work within the broader policy debate on crime control. Our findings suggest that policies emphasizing early intervention, targeted rehabilitation efforts, and a reconsideration of first-time arrest practices may be more effective in reducing long-term crime disparities than punitive measures alone.

The remainder of this paper proceeds as follows. We begin with a brief review of the literature, including past criminological work that has invoked a systems approach, the criminological constructs that anchor our model, and literature on theories of persistent racial disparities in crime. We then present and analyze our dynamic systems model. Next, we calibrate our model to empirical data on self-reported offending and arrests from NLSY97 and estimate the transition probabilities from our dynamic system model. We then demonstrate how marginal changes in flows between states (as described in our dynamic systems model) ultimately influence the long-run level of crime and demographic differences in criminal involvement. After summarizing the contributions, limitations and assumptions behind our analyses, the final section concludes.

## Relevant literature

Although crime and criminal justice have long been understood as complex systems with interconnected components [1], a great deal of work in criminology has focused on the effects of interventions on separate components in isolation—explicitly regarding other components as confounders to be controlled. These “reduced form," regression-based approaches are crucial for learning the value of a causal effect but can miss the interconnected mechanisms that produce it. This can lead to mixed results in empirical literatures and to unintended consequences for public policy.

For example, the evidence on the effectiveness of arrest in reducing crime is quite mixed. Although it is clear that police presence and the threat of arrest carry a general deterrent effect [2], some evidence suggests that arrest itself can lead to higher crime rates [3], particularly for Black and Latino boys [4]. This is consistent with labeling processes [5] that disproportionately impact Black and Latino boys, with psychological distress, and with cumulative disadvantage [6] such as issues in finding employment with a criminal record. A dynamical systems model can endogenize these considerations in a dynamic setting, uncovering conditions under which a given arrest policy would raise or lower overall crime—and revealing which parameters need to be more reliably estimated (with reduced form approaches) for the appropriate policy recommendation. In this way, systems modeling is not a replacement for reduced-form approaches but a complement. Together, both are made more productive. Instead of solely focusing on a single components of what is truly an integrated system, solving systemic problems requires a systems approach.

### Systems approach

To motivate our dynamic systems methodology, we begin with an overview of the literature on systems approaches to modeling the dynamics of crime. A systems approach involves the explicit modeling and analysis of a complex interdependent system. This allows us to identify “high leverage" points, simulate potential unintended policy consequences, and systematically enlighten academic debates that may be frustrated by a complex system’s counterintuitive behavior.

Systems modeling in criminology began with the seminal work of Alfred Blumstein, who served on the President’s Commission on Law Enforcement and Administration of Justice in 1967. Blumstein helped demonstrate how such models can project the operating costs of various components of the criminal justice system and how interventions on the various components or demographic shifts in the population influence overall crime rates [7].

A related literature, evolving in parallel, was spurred by Becker [8], who treated criminal activity as a rational economic choice in a general equilibrium model. This led more broadly to the subfield known as the “economics of crime" [9], which generally involves bottom-up approaches in which researchers specify the decision rules of individual criminals, including explicit consideration of the potential deterrent effect of the threat of punishment. For example, economists have used this decision-based framework to describe equilibrium levels of crime by neighborhood characteristics,[10, 11], and to determine optimal allocation of police forces [12].

Others have used operations research techniques to model the recidivism process [13–18], for example, to capture “the feedback into society of offenders released at various stages in the system” [19]. At the micro level, Blumstein and colleagues [20],[21] analyzed trajectories of individual participation in crime and estimated an individual-level rate of offending and how it changed over time. This led to explorations of “selective incapacitation,” focusing on individuals who would have relatively higher rates of offending were they not behind bars [22, 23]. On the other hand, Durlauf and Nagin [24] challenged the evidence that incarceration is a major deterrent of crime.

Related quantitative models of the spread of crime include Blumstein’s diffusion model [25] with its focus on the diffusion of criminal activity resulting from the introduction of crack cocaine into cities. Blumstein [26] further argued that the replacement of incarcerated drug offenders led to further diffusion of drug and gun crimes. Short and colleagues [27–29] used agent-based models and reaction-diffusion differential equations to characterize “hotspots” of criminal activity that might call for increased police vigilance.

A number of authors have employed game-theoretic approaches with interacting agents. Perc and coauthors show that under certain conditions, increasing punishment could actually increase crime levels [30]. Berenji and coauthors study the effects of incarceration and prisoner reentry interventions on recidivism using an evolutionary game, finding an optimal mix of punishment and rehabilitation efforts is particularly effective for those returning from prison for the first time [31]. Wang and coauthors derive the equilibrium amount of criminal activity in a neighborhood when agents have heterogeneous opportunity costs that depend on the level of crime in a neighborhood [32].

See [33] for more details on these and other quantitative approaches to the spread of crime.

Our prior papers [33, 34] use dynamical systems models to study the spread of crime. These models can be written as a system of differential equations that represent the flow into and out of various states of criminal activity, in terms of certain fixed “transition parameters." In that work, we presented a theoretical model for the spread of crime using parameters that capture the stocks and flows through key states in the life course, including initiation into crime, incarceration, recidivism, desistance, and rehabilitation. In one paper [33], we derived theoretical conditions that lead to high vs low-crime equilibria and found that increasing the incarceration rate of first-time offenders can actually increase the level crime in the system if recidivism is large compared with successful post-incarceration rehabilitation. A follow-up paper allowed for more heterogeneous agents, especially by age—a step that required use of agent-based models [34].

Together, these papers have been followed by a body of work on dynamic models of the spread of crime [35–38], violent extremism [39], terrorist networks [40], radicalization [41], criminal gangs [42], and the role of educational programs in deterring crime [43].

In this paper, we take the critical next step of using empirical data to inform and extend our earlier theoretical models. We use data from the National Longitudinal Survey of Youth 1997 cohort (NLSY97) to estimate the transition parameters of our dynamical systems model. We include separate transition parameters for three racial groups, referred to in the NLSY surveys as “Black," “Hispanic," “White" and two sexes identified in the surveys as “male" and “female." We also present a new theoretical model of crime dynamics that revises our prior work by focusing on arrest rather than incarceration as the initial societal response to crime, with separate states for returning to or abandoning criminal activity after arrest. We studied contagion theoretically in previous work [33] but will not consider it in this paper. Instead, we will focus on a linear dynamical system with easily empirically identifiable parameters so that we can not only quantify the parameters but also pin down the conditions that lead to certain theoretical conclusions. For example, do the parameter estimates suggest that increasing first-time arrest rates would increase or decrease crime? And does this vary by subgroup? These questions would not be answerable in a regression context because the effect of first-time arrest on long-run crime is endogenous to the effects of other flow rates (e.g., desistance and recidivism rates). We discuss the limitations and assumptions of the dynamical systems approach in the final section.

### Racial disparities

A central focus of this paper is to understand how racial differences in certain transition rates in and out of states of criminal activity interactively generate long-run disparities in crimes rates. Black Americans are more likely to be arrested for the same offense compared to Whites [44]. Compared to Whites, Black Americans who have been arrested receive harsher sentencing [45] and are more likely to be victims of police brutality [46, 47].

Explanations of racial disparities in crime and criminal justice have long been debated in academic and political circles [48, 49]. Mallet and coauthors [50] suggest that there are at least four dominant theories to explain the overrepresentation of minorities in the criminal justice system: larger offense rates among minorities, racial bias in decision-making among criminal justice officials, a disproportionate accumulation of individual risk factors such as neglect and mental health problems, and contextual differences (such as disparities in school quality or communities with fewer employment opportunities). Rehavi and Starr [45] showed that, on average, Blacks receive almost ten percent longer sentences than comparable Whites for the same crimes and that at least half of this gap can be explained by differences in initial charging choices of prosecutors. Yet, Blumstein [51] attributed racial disparities in U.S. prisons chiefly to differences in arrest rates rather than racial bias in the criminal justice system. These arrest differences could be driven by differences in policing or offense rates and, in the latter case, the offense rates could be partially explained by racial barriers to education attainment. Lochner and Moretti [52] found that an additional year of schooling is associated with 3 to 4 percent decrease in likelihood of incarceration for Whites, an 8 to 9 percent decrease for Blacks, and an 11 percent reduction in the likelihood of arrest, with the effect being larger for Blacks than Whites.

While scholars disagree on the causes of racial gaps in criminal involvement, most agree that intergroup disparities in crime and criminal justice involve several possible, mutually interacting components. Racial disparities in different components of these systems can become mutually reinforcing—a major property of what has become known as “systemic discrimination" [53, 54]. If the dynamics of crime involve a time-varying, state-dependent process whereby past behavior alter the trajectory of future behavior [55], then static models to explain racial disparities in crime will miss how differences in flows between states interact to dynamically magnify the disparities we observe. However, there is a longstanding debate on the extent to which the correlation between past and future offending is due to a state-dependent process, or time-invariant, person-specific traits that drive population heterogeneity in the propensity to commit crimes [55]. Our analysis includes a random intercepts specification designed to estimate our parameters “net of" such population heterogeneity, so that we can focus our simulations on the consequences of state-dependent criminal dynamics.

Our approach sheds light on the dynamics of racial disparities in long-run crime levels, including how differences in likelihoods of important parameters (e.g., rates of arrest of first-time vs. repeat offenders, rehabilitation, initiation, and desistance) interactively generate long-run disparities. Perhaps even more importantly, devising a crime control strategy in a world of state-dependent criminality requires that we understand how independent changes in one pathway of intervention could have reinforcing or counter-productive effects along other pathways. Our approach can be useful to this end.

### The dynamics of crime through the life course

We now review the criminological literature relevant to the components of our dynamic systems model. During the life course, people can experience several different kinds of transitions related to criminal involvement. Initial participation in crime, or criminal “onset" [56], can mark the beginning of a long or short-lived “criminal career" [57], characterized by periods of undetected activity, first-time arrest (primary detection), repeat-arrest (secondary detection), recidivism, and desistance from crime. Each of these features of criminal dynamics have usually been studied as separate outcomes to be explained, or as isolated causal variables in and of themselves. This paper will study how they jointly and interactively influence long-run crime.

Initial criminal onset is impacted by a wide range of individual, social, and economic factors, including but not limited to punitive school disciplinary policies that leave racialized impacts [58, 59], psychological hardship related to adverse childhood experiences [60], and victimization [61]. Undetected criminal activity is generally measured through victimization reports or self-reports. The 1997 National Longitudinal Survey of Youth allows us to link self-reports of crime with arrest records and thus to measure both detected and undetected initial criminal onset of youth.

A growing body of research views criminal justice contact, especially first arrest, as an important turning point in the criminal life course [3, 4, 62]. In certain contexts, arrest can increase crime by destabilizing conflict between groups [63] or by labeling [5, 64]. For example, the adoption of a societally imposed identity as a “deviant" individual, a criminal record, and a resultant lack of opportunity can make it difficult to be rehabilitated back into society. The notion that the initial arrest can have a reinforcing impact on subsequent crime stands in contrast to both the “specific deterrent" effect of arrest, through which the experience of punishment is believed to decrease subsequent crime [65, 66], and the “general deterrent" effect of the threat of future punishment [2, 8, 67]. Both theoretically and empirically, the effect of arrest on recidivism is ambiguous—an ambiguity our model attempts to explain with systems thinking, taking seriously the possibility of first arrest as a “turning point." Less ambiguous are the cumulative psychological, social, and economic disadvantages that drive recidivism, including labor market discrimination, persistent poverty, and social isolation [6, 68].

Predictors of desistance from crime include employment, social supports, marriage, reproduction, and other important life events [6, 69, 70]. Desistance appears to be more likely for women (especially when having children) [71] and less likely for Black people. To our knowledge, our paper is the first to demonstrate that, among those with no arrest history, Black women have significantly higher annual desistance rates than Black, White, or Hispanic men or women. Temporary desistance is sometimes contrasted with complete cessation from crime, which we refer to in this paper as rehabilitation—distinct from, though informed by, the criminological concept of reintegrating former prisoners into society [72].

The regression-based approaches employed in most of this work has taught us a great deal about what drives each of these aspects of crime dynamics. We understand what policy variables might influence criminal onset rates, first-time vs. repeat arrest rates, undetected recidivism rates, desistance, and rehabilitation. But each of these components generates long-run crime levels in an interdependent fashion. Which of these aspects of the criminal life course involve the greatest intergroup disparities? Were we to intervene appropriately, which would yield the largest decreases in long-run crime, given the others? How do the answers to these questions depend on the true empirical value of these parameters? This paper addresses these questions using a dynamical systems approach.

## The model

### Modeling overview

In this section we present a mathematical model of the flow of populations in and out of states of criminal activity and arrest. A summary of the assumptions and limitations of the model is provided in the final section. Given the model’s assumptions, we now analyze how the parameters of the model interactively lead to long-run equilibrium levels of crime.

**Fig 1:**
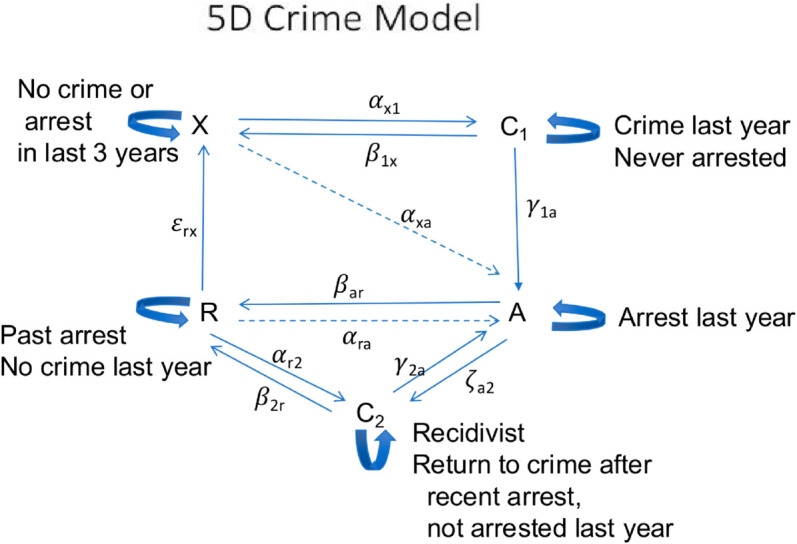
Flow diagram for the 5D model.

Our five-dimensional model arose from a simpler three-dimensional model. Because the mathematical analysis of the 5D-model flows naturally from the mathematical analysis of the 3D-model, we present and work with the simpler 3D-model in the Supporting information [S1 Fig: S1 File]. The 3D-model misses a critical piece of the dynamic—the distinction between those with and without an arrest history. An effective model needs to keep track of those who returned to crime after arrest and those who avoided criminal behavior after their arrest. Fig 1 below summarizes these states and flows.

### States in the model

The five states $X,C_{1},A,R,C_{2}$ in the model are defined as follows:

*X* Those who either
  1) have never been arrested, and have not offended in the last year, or
  2) have been arrested before, but have not offended in the last three years (rehabilitation).
*C*_1_ Those who have never been arrested before, but have offended in the last year.
*A* Those who have been arrested in the last year, regardless of self-reported offending.
*R* Those who have been arrested or have offended in the last three years, but not in the last year.
*C*_2_ Those who
  1) have been arrested before but not in the last year and
  2) have offended in the last year.

### Transitions in the model

- Transitions from *X*
  - No crime in last year: $\sigma_{xx}:X\to X$.
  - Crime in last year, no arrest: $\alpha_{x1}:X\to C_{1}$ (onset)
  - Crime and arrest in last year: $\alpha_{xa}:X\to A$ (onset)
- Transitions from *C*_1_
  - No crime in last year and no arrest in last year: $\beta_{1x}:C_{1}\to X$. (desistance)
  - Crime in last year, but no arrest: $\sigma_{11}:C_{1}\to C_{1}$.
  - Arrest (and crime) in last year: $\gamma_{1a}:C_{1}\to A$. (first-time arrest)
- Transitions from *A*
  - No crime in last year: $\beta_{ar}:A\to R$:.
  - Crime and arrest in last year: $\sigma_{aa}:A\to A$.
  - Crime, but no arrest in last year: $\zeta_{a2}:A\to C_{2}$.
- Transitions from *C*_2_
  - Crime and arrest in last year: $\gamma_{2a}:C_{2}\to A$.
  - Crime, but no arrest last year: $\sigma_{22}:C_{2}\to C_{2}$.
  - No crime last year: $\beta_{2r}:C_{2}\to R$.
- Transitions from *R*
  - Crime last year, no arrest: $\alpha_{r2}:R\to C_{2}$ (recidivism)
  - Crime and arrest last year: $\alpha_{ra}:R\to A$.
  - No crime last year, but crime in last three years: $\sigma_{rr}:R\to R$.
  - No crime in last three years: $\epsilon_{rx}:R\to X$. (rehabilitation)

The transition parameters within each of the above five bullets must sum to 1. The dynamic system can be described with the following set of difference equations:


$$X(t+1)=\sigma_{xx}X(t)+\beta_{1x}C_{1}(t)+\epsilon_{rx}R(t)$$

$$C_{1}(t+1)=\sigma_{11}C_{1}(t)+\alpha_{x1}X(t)$$

$$A(t+1)=\sigma_{aa}A(t)+\gamma_{1a}C_{1}(t)+\alpha_{xa}X(t)+\alpha_{ra}R(t)$$

$$C_{2}(t+1)=\sigma_{22}C_{2}(t)+\zeta_{a2}A(t)+\alpha_{r2}R(t)$$

$$R(t+1)=\sigma_{rr}R(t)+\beta_{2r}C_{2}(t)+\beta_{ar}A(t)$$

Because the transition probabilities from each of the five states add to 1, we can replace the *σ*s in this system and, after simplifying, rewrite it as:


$$X(t+1)-X(t)=\beta_{1x}C_{1}+\epsilon_{rx}R-\alpha_{x1}X-\alpha_{xa}X$$

$$C_{1}(t+1)-C_{1}(t)=\alpha_{x1}X-\beta_{1x}C_{1}-\gamma_{1a}C_{1}$$

$$A(t+1)-A(t)=\gamma_{1a}C_{1}+\alpha_{ra}R+\gamma_{2a}C_{2}+\alpha_{xa}X-\beta_{ar}A-\zeta_{a2}A$$ (1)

$$R(t+1)-R(t)=\beta_{ar}A+\beta_{2r}C_{2}-\alpha_{r2}R-\alpha_{ra}R-\epsilon_{rx}R$$

$$R(t+1)-R(t)=\beta_{ar}A+\beta_{2r}C_{2}-\alpha_{r2}R-\alpha_{ra}R-\epsilon_{rx}R$$

Just as for our 3D system, the right hand sides of system (1) sum to 0; population has constant size $N=X(t)+C_{1}(t)+A(t)+R(t)+C_{2}(t).$

### Equilibrium

We can therefore eliminate one variable and one equation from (1) and work with a linear system of four equations in four unknowns. Furthermore, as we did for the 3D model, we can divide each of these equations through by *N* so that the five variables become population fractions instead of total numbers. Finally, as we did in the 3D system, we set the right hand sides of system (1) equal to zero to compute the long run equilibrium to which the dynamics flow from any starting (t=0) values of the variables. As we explain in the Supporting Information [Supporting Information: S1 File], the long run equilibrium value for *X* is:

$$X^{*}=\frac{\epsilon_{rx}(\beta_{1x}+\gamma_{1a})(\gamma_{2a}\beta_{ar}+\beta_{2x}(\beta_{ar}+\zeta_{a2}))}{D}$$ (2)

where the denominator is:


$$D=\{\alpha_{x1}[\epsilon_{rx}\gamma_{1a}\gamma_{2a}+\epsilon_{rx}\gamma_{2a}\beta_{ar}+\gamma_{1a}\gamma_{2a}\beta_{ar}+\gamma_{1a}\gamma_{2a}\alpha_{ra}\;\;+\epsilon_{rx}\gamma_{1a}\zeta_{a2}+\gamma_{1a}\alpha_{ra}\zeta_{a2}+\alpha_{x2}\gamma_{1a}(\gamma_{2a}+\beta_{ar}+\zeta_{a2})\;\;+\beta_{2x}(\epsilon_{rx}(\gamma_{1a}+\beta_{ar}+\zeta_{a2})+\gamma_{1a}(\beta_{ar}+\alpha_{ra}+\zeta_{a2}))]\;\;+(\beta_{1x}+\gamma_{1a})[\epsilon_{rx}(\gamma_{2a}(\beta_{ar}+\alpha_{xa})+\alpha_{xa}\zeta_{a2})+\alpha_{xa}(\gamma_{2a}(\beta_{ar}+\alpha_{ra})+\alpha_{ra}\zeta_{a2}+\alpha_{x2}(\gamma_{2a}+\beta_{ar}+\zeta_{a2}))\;\;+\beta_{2x}(\epsilon_{rx}(\beta_{ar}+\alpha_{xa}+\zeta_{a2})+\alpha_{xa}(\beta_{ar}+\alpha_{ra}+\zeta_{a2}))]\}.$$

### Effect of parameter changes on X^*^

This long run equilibrium level of crime depends on the underlying dynamic parameters. Roughly speaking, if the effect of a particular intervention can be interpreted as an independent change in one of the parameters in our model, we investigate how the level of criminal activity will be affected by that change. We again focus on X^*^, the fraction of those in the population who have not had any criminal activity or have not been arrested at least for the past three years. We loosely call $1-X^{*}=C_{1}^{*}+A^{*}+C_{2}^{*}+R^{*}$ the “crime rate.”

None of the *α*s appears in the numerator of X^*^. Thus, by the quotient rule, $\frac{\partial X^{*}}{\partial \alpha }$ is <0 for all four *α*s. This leads to the obvious conclusion that an increase in the rate of entry or reentry into crime leads to a decrease in X^*^ and an increase in the crime rate.

It takes a little more work to show that


$$\frac{\partial X^{*}}{\partial \beta_{1x}}>0,\;\frac{\partial X^{*}}{\partial \beta_{2r}}>0,\;\frac{\partial X^{*}}{\partial \beta_{ar}}>0,\;\frac{\partial X^{*}}{\partial \epsilon_{rx}}>0,$$

as it did in the 3D model [Supporting Information: S1 File]. We conclude that increasing the rate of movement into *R* or back to *X* decreases the crime rate.

The effects of an increase in the arrest rates are more subtle. For re-arrests, the derivative is:


$$\frac{\partial X^{*}}{\partial \gamma_{2a}}=\frac{\epsilon_{rx}(\beta_{1x}+\gamma_{1a})(\beta_{ar}-\beta_{2r})[\alpha_{x1}\gamma_{1a}+(\beta_{1x}+\gamma_{1a})\alpha_{xa}][(\epsilon_{rx}+\alpha_{ra})\zeta_{a2}+\alpha_{r2}(\beta_{ar}+\zeta_{a2})]}{D^{2}}\;\;$$

The sign of this derivative depends on $\beta_{2r}$ and $\beta_{ar}$. Specifically, the derivative is nonnegative if $\beta_{ar}\geq \beta_{2r}$ and negative otherwise. Simply put, arresting criminals with an arrest history reduces crime if they are more likely to desist after arrest than before arrest.

The derivative for first arrest is:


$$\frac{\partial X^{*}}{\partial \gamma_{1a}}=\frac{\alpha_{x1}\epsilon_{rx}(\gamma_{2a}\beta_{ar}+\beta_{2r}(\beta_{ar}+\zeta_{a2}))}{D^{2}}\times [\epsilon_{rx}[\gamma_{2a}\beta_{ar}+\beta_{2r}(\beta_{ar}+\zeta_{a2})]\;\;\;\;\;\;-\beta_{1x}[\gamma_{2a}(\epsilon_{rx}+\beta_{ar}+\alpha_{ra})+\zeta_{a2}(\epsilon_{rx}+\alpha_{ra})+\alpha_{r2}(\gamma_{2a}\;+\beta_{ar}+\zeta_{a2})+\beta_{2r}(\epsilon_{rx}+\beta_{ar}+\alpha_{ra}+\zeta_{a2})]].$$

Its sign depends on the relative size of $\epsilon_{rx}$, the rehabilitation rate of those with an arrest history, to $\beta_{1x}$, the desistance rate of those never arrested. In short, if people are more likely to desist before they have any arrest history than after they obtain an arrest history, then increasing the arrest rate of the never-arrested can increase the overall crime rate.

Finally, our analysis indicates that the first and second derivatives of X^*^ are always opposite in sign, which implies a decreasing returns effect of each parameter on the equilibrium level X^*^. For example, as $\beta_{1x}$ increases, its positive impact on X^*^ decreases; and as $\alpha_{x1}$ increases, its negative impact on X^*^ decreases.

### $\alpha_{xa}:X\to A$ is a complex, but important transition

Transition parameters $\alpha_{xa}:X\to A$ plays a particularly important role. This is a complex parameter in that it includes two changes coming in one year: onset of criminal activity ($\alpha_{x1}:X\to C_{1}$) and first arrest ($\gamma_{1a}:C_{1}\to A$) in the same time step. It represents a transition in which first arrest occurs very close in time to the onset of criminal offending.

With this theoretical analysis in mind we turn to the data used to calibrate the model.

## Data: NLSY97

We empirically estimate parameter values in our mathematical models using data from the National Longitudinal Survey of Youth 1997 cohort (NLSY97), a representative sample of people born between 1980 and 1984 and living in the United States at the time of the initial survey in 1997. We use data from annual interviews conducted from 1997 to 2003 (Rounds 1-7), when questions about self-reported criminal activity were asked. Our analytic sample consists of 6,459 who responded to all seven survey waves and who identify with NLSY racial descriptors “Black," “White," or “Hispanic." There were 8,984 interviewees in 1997; 7,754 of these were still being interviewed in 2003. We dropped 1,295 of these individuals who did not respond to all seven survey waves or who did not identify as Black, White, or Hispanic, leaving our final sample size of 6,459. We note that the NLSY97 interviews incarcerated individuals, but that in the NLSY97, incarceration is a rare event. Across all 45,213 observations in our analytic sample, only 0.73% (330) involved incarcerated individuals. This is close to the 0.9% (520) of 57,134 observations that involved incarcerated individuals in the broader sample.

To operationalize the states from our theoretical model, we used data from self-administered survey questions about participation in criminal and delinquent activity as well as arrests. We consider someone to have committed an offense in the past year if they reported being involved in any of the following activities within the last year: purposely damaging or destroying property not belonging to the respondent, stealing something worth less than $50, stealing something worth $50 or more (including a car), other property crimes (including fencing stolen property, possessing or receiving stolen property, or selling something for more than it was worth), or attacking or assaulting someone. To measure arrests, we used data on whether the respondent had been arrested by the police or taken into custody for an illegal or delinquent offense (not including arrests for minor traffic violations) within the last year. Using these self-reports of criminal activity and arrests, we constructed binary variables for each of the five states of our theoretical model, using the formal definitions presented earlier. For example,

- *X* (criminally inactive, no recent arrest history) = 1 if the respondent (1) has never been arrested and not offended within the last year or (2) has been arrested but has not offended or been arrested within the last three years. We note that in our data, less than 0.5% of the sample self-reports crime when they have desisted for more than three years. We consider those inactive for more than three years “rehabilitated."
- *C*_1_ (criminally active, never arrested) = 1 if the respondent (1) has never been arrested but (2) has offended within the last year;
- *A* (arrested) = 1 if the respondent has been arrested within the last year, regardless of offense activity;
- *C*_2_ (criminally active, recent arrest history) = 1 if the respondent (1) has been arrested but not within the last year and (2) has offended in the last year;
- *R* (criminally inactive with recent arrest history) = 1 if the respondent (1) reported an arrest in a previous survey wave, (2) has offended or been arrested within the last three years, but (3) has neither offended nor been arrested in the last year.

Individuals in our sample are classified into one of these mutually exclusive states at each survey wave but over time they may move in and out of different states. Our primary outcomes for the statistical analysis presented below are wave-to-wave transitions between different states in our model, as illustrated by the arrows in Fig 1.

The sixteen transitions are carefully delineated in the Supporting Information [Supporting Information: S1 File.] For example,

- someone who was criminally inactive in the previous wave and remains inactive in the following wave is assigned the X→X transition,
- someone who was criminally inactive in the previous wave but reports involvement in criminal offending at the next wave without arrest is assigned the $X\to C_{1}$ transition.

We analyze differences in these transitions by sex, age, and race/ethnicity, using data on sex (men/women) and race/ethnicity (non-Hispanic Black, non-Hispanic White, and Hispanic) collected at Round 1 of NLSY97, and a time-varying measure of age. Our analytic sample is 51% men and 49% women, 26% non-Hispanic Black, 51.9% non-Hispanic White, and 21.2% Hispanic. The mean age at baseline (Round 1) was 14.9 years.

## Methods

### Assignment to model state in each round

Our analysis proceeds in three stages. First, we present descriptive results showing trends over time and across subgroups in the proportional sizes of the five compartments of our theoretical model. We also present a transition matrix showing flows between each of the compartments in our theoretical model from one survey wave to the next.

**Fig 2:**
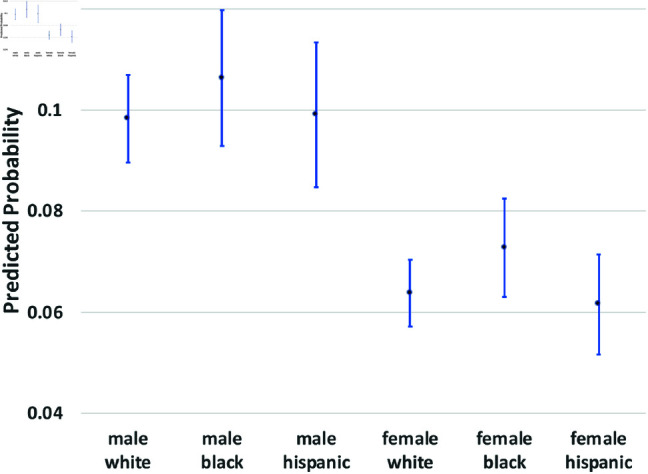
$X\to C_{1}$ by Subgroup.

Second, we analyze differences across demographic groups in each of the 16 possible transitions in our theoretical model by running logistic regression models with random effects, using the following specification:


$$\log\left[\frac{\text{Pr}(y_{i(t\to t+1)}=1)}{1-\text{Pr}(y_{i(t\to t+1)}=1)}\right]=\beta_{0}+\beta_{1}\;\text{(Age}-16{)}_{it}+\beta_{2}\;\text{(Age}-16{)}_{it}^{2}+\beta_{3}\;{\text{Woman}}_{i}\;+\beta_{4}\;{\text{Black}}_{i}+\beta_{5}\;{\text{Hispanic}}_{i}+\beta_{6}\;{\text{(Woman}}_{i}*{\text{Black}}_{i})+\beta_{7}\;({\text{Woman}}_{i}*{\text{Hispanic}}_{i})+u_{i}$$

where $y_{i(t\to t+1)}$ is a binary outcome indicating the transition between states in our theoretical model from survey wave *t* to wave t+1. Since our study period consists of seven waves of NLSY data, we observe six wave-to-wave transitions for each person. We control for age as a quadratic function, but the main coefficients of interest are the dummy variables for sex, race/ethnicity, and the interactions between them. The model also includes a random effect, *u*_*i*_, which is assumed to be marginally normal. The purpose of this error term is to proxy for person-specific, time-invariant traits, such as cultural background, childhood personality traits, and (possibly) a “natural” propensity to commit crimes. Although this approach does not fully account for such population heterogeneity concerns, it does address them. Using these regression models, we calculate the average predicted probabilities for subgroups defined by the interaction between sex and race/ethnicity—White men and women, Black men and women, and Hispanic men and women, and we graph these predicted probabilities along with their 95% confidence intervals in Figs 2 through 12.

**Fig 3:**
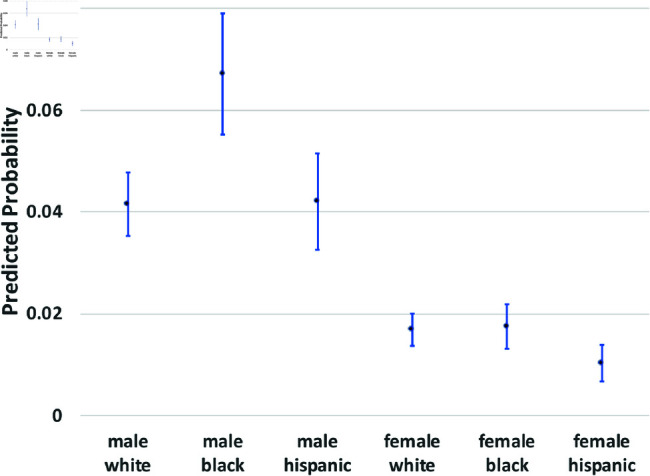
X→A by Subgroup.

**Fig 4:**
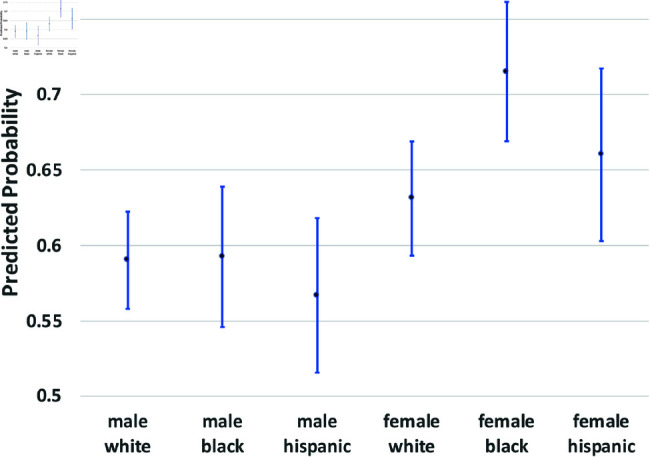
$C_{1}\to X$ by Subgroup.

**Fig 5:**
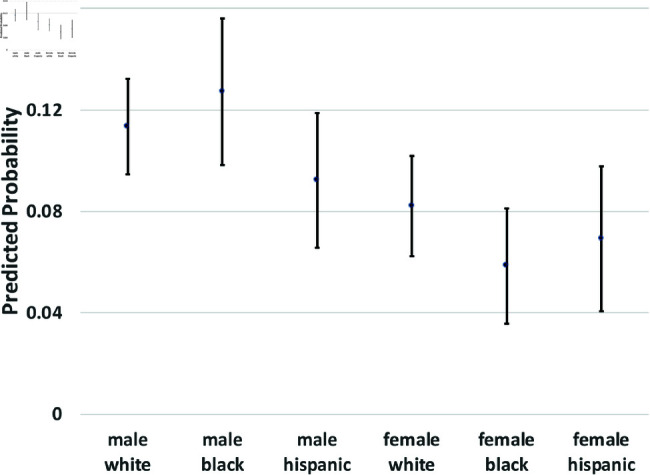
$C_{1}\to A$ by subgroup.

**Fig 6:**
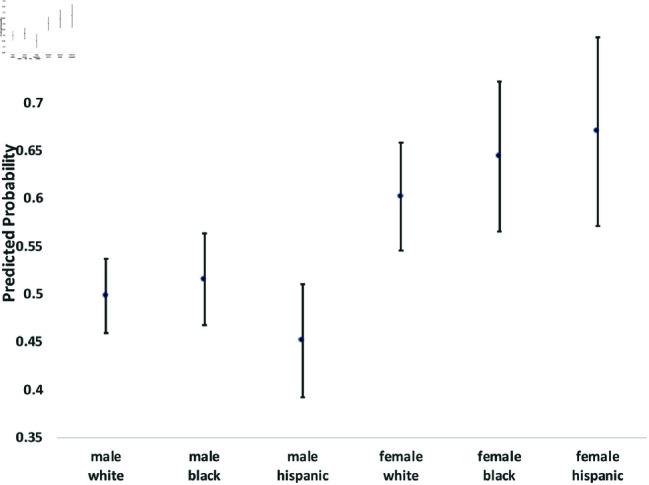
A→R by Subgroup.

**Fig 7:**
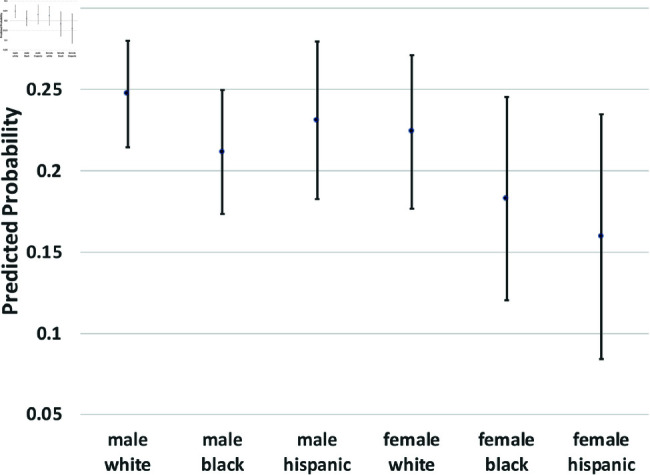
$A\to C_{2}$ by Subgroup.

**Fig 8:**
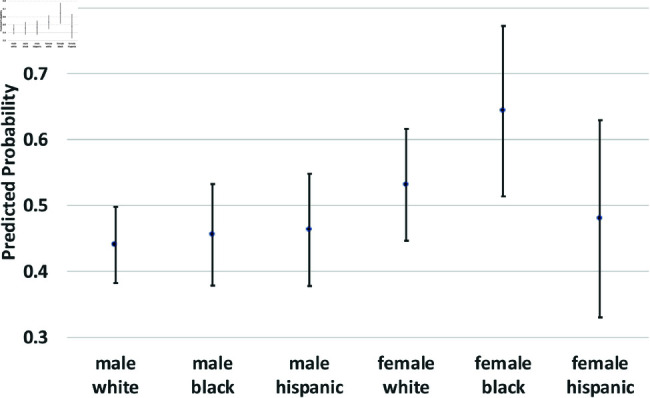
$C_{2}\to R$ by Subgroup.

**Fig 9:**
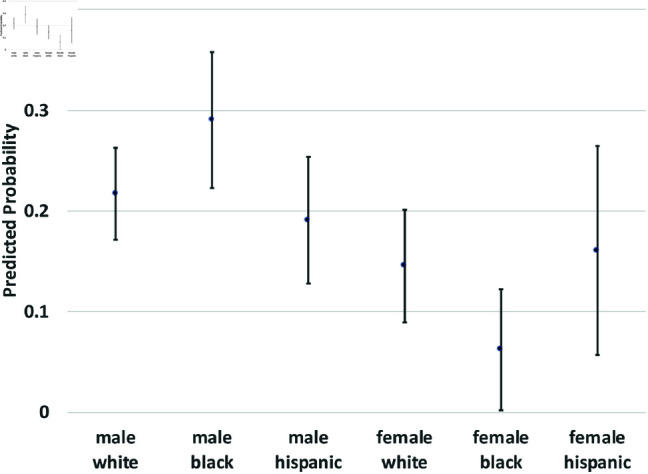
$C_{2}\to A$ by Subgroup.

**Fig 10:**
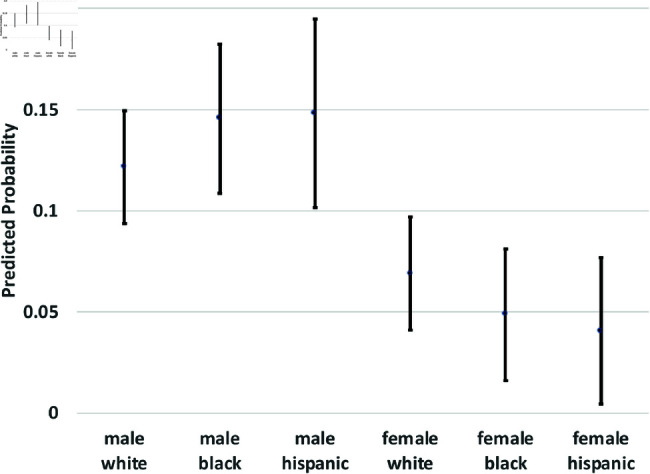
R→A by Subgroup.

**Fig 11:**
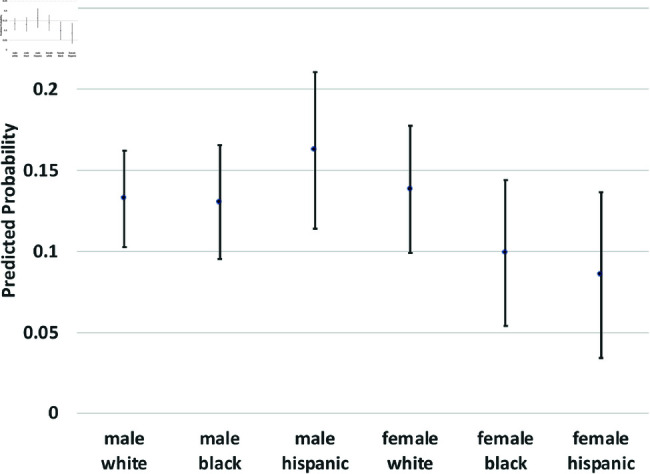
$R\to C_{2}$ by Subgroup.

**Fig 12:**
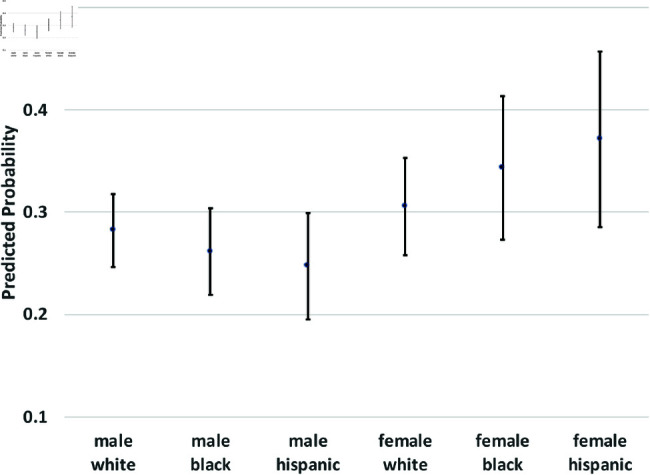
R→X by Subgroup.

Third, we carry out simulations to analyze how each of the transition probabilities affect the long-run equilibrium of the system. For each of the six subpopulations, and then for the total population, we substitute the estimated values of the transition parameters from Figs 2–12 for that subpopulation into dynamical system (1). Then, for each subpopulation, we use the Round 1 survey values from Table 1 to set the initial values $X(0),C_{1}(0),A(0),C_{2}(0),R(0)$ of each of the five states. We simulate system (1) to calculate the corresponding values at *t* = 1 from the values at *t* = 0, and so on for t=2,3,4,…. This linear process converges to a unique, stable “long run" equilibrium state distribution regardless of initial conditions. Expression (2) provides a formula for this equilibrium X^*^ for state *X* for any choice of the transition parameters. We show how quickly convergence occurs, and that this is indeed the distribution to which the system dynamics converge.

**Table 1:** 5D Model compartments by year and demographic group.

	X	$C_{1}$	A	R	$C_{2}$	Total
Full Sample						
Round 1 (1997)	0.855	0.079	0.041	0.013	0.012	1.000
Round 2 (1998)	0.765	0.136	0.052	0.021	0.025	1.000
Round 3 (1999)	0.760	0.113	0.054	0.025	0.048	1.000
Round 4 (2000)	0.764	0.090	0.056	0.029	0.061	1.000
Round 5 (2001)	0.771	0.070	0.059	0.031	0.069	1.000
Round 6 (2002)	0.779	0.054	0.057	0.029	0.081	1.000
Round 7 (2003)	0.798	0.041	0.046	0.028	0.086	1.000
White Men						
Round 1 (1997)	0.827	0.094	0.044	0.017	0.017	1.000
Round 2 (1998)	0.733	0.147	0.064	0.025	0.031	1.000
Round 3 (1999)	0.722	0.121	0.074	0.030	0.054	1.000
Round 4 (2000)	0.708	0.107	0.078	0.037	0.071	1.000
Round 5 (2001)	0.721	0.073	0.073	0.035	0.098	1.000
Round 6 (2002)	0.717	0.059	0.084	0.044	0.096	1.000
Round 7 (2003)	0.741	0.050	0.059	0.036	0.114	1.000
Black Men						
Round 1 (1997)	0.796	0.092	0.074	0.025	0.014	1.000
Round 2 (1998)	0.670	0.161	0.094	0.030	0.045	1.000
Round 3 (1999)	0.654	0.118	0.110	0.032	0.087	1.000
Round 4 (2000)	0.653	0.084	0.112	0.045	0.106	1.000
Round 5 (2001)	0.647	0.064	0.127	0.048	0.115	1.000
Round 6 (2002)	0.638	0.056	0.110	0.041	0.155	1.000
Round 7 (2003)	0.656	0.038	0.103	0.054	0.149	1.000
Hispanic Men						
Round 1 (1997)	0.786	0.106	0.073	0.018	0.018	1.000
Round 2 (1998)	0.698	0.142	0.091	0.035	0.034	1.000
Round 3 (1999)	0.682	0.125	0.080	0.039	0.075	1.000
Round 4 (2000)	0.676	0.100	0.084	0.043	0.097	1.000
Round 5 (2001)	0.701	0.074	0.089	0.048	0.088	1.000
Round 6 (2002)	0.699	0.066	0.074	0.043	0.118	1.000
Round 7 (2003)	0.732	0.036	0.083	0.035	0.114	1.000
White Women						
Round 1 (1997)	0.902	0.053	0.027	0.007	0.011	1.000
Round 2 (1998)	0.828	0.103	0.035	0.014	0.020	1.000
Round 3 (1999)	0.828	0.089	0.031	0.012	0.041	1.000
Round 4 (2000)	0.849	0.059	0.031	0.020	0.042	1.000
Round 5 (2001)	0.847	0.057	0.032	0.018	0.046	1.000
Round 6 (2002)	0.864	0.038	0.030	0.014	0.054	1.000
Round 7 (2003)	0.882	0.028	0.022	0.013	0.054	1.000
Black Women						
Round 1 (1997)	0.878	0.074	0.029	0.009	0.010	1.000
Round 2 (1998)	0.823	0.109	0.028	0.009	0.031	1.000
Round 3 (1999)	0.811	0.108	0.025	0.016	0.040	1.000
Round 4 (2000)	0.848	0.064	0.034	0.012	0.042	1.000
Round 5 (2001)	0.853	0.053	0.033	0.014	0.047	1.000
Round 6 (2002)	0.876	0.035	0.024	0.011	0.054	1.000
Round 7 (2003)	0.882	0.032	0.021	0.016	0.050	1.000
Hispanic Women						
Round 1 (1997)	0.889	0.065	0.026	0.010	0.010	1.000
Round 2 (1998)	0.822	0.117	0.024	0.011	0.026	1.000
Round 3 (1999)	0.868	0.058	0.025	0.011	0.039	1.000
Round 4 (2000)	0.861	0.066	0.019	0.017	0.037	1.000
Round 5 (2001)	0.881	0.045	0.023	0.013	0.039	1.000
Round 6 (2002)	0.888	0.040	0.022	0.014	0.037	1.000
Round 7 (2003)	0.900	0.032	0.026	0.009	0.033	1.000

To study how sensitive the equilibrium state distribution is to changes in the parameters, we simulate how X^*^ is affected by changes in each of the transition probabilities. Our simulations show how sensitive the value of X^*^ is to a one-percentage-point change in each parameter, which we compare to the partial derivatives computed earlier. Our simulations also show how sensitive X^*^ is to larger changes in each parameter.

Finally, we use the simulations to analyze how racial differences in transition probabilities contribute to racial differences in the equilibrium level of crime. Our final task in this vein was to analyze which parameter(s) played the largest role in Black/White differences. We first computed and examined the absolute and relative differences in the transition parameters for Black males and White males; for the relative differences, we used the quotient: (Black rate – White rate)/White rate. Finally, we considered the possible systems-level effects behind some of these racial differences. Could the *complex system* of interrelated transitions cause long-run crime differences to be more sensitive to certain parameters than others, regardless of the size of the racial differences in those transition parameters? To shed light on this question, we ran our dynamic simulation (1) with the transition parameters for White males with one exception—we changed one parameter to the corresponding value for Black males. We repeated this for each parameter. Then we reversed the process, using all but one parameter from the Black male list and replacing that parameter by the corresponding parameter from the White male list.

## Results for state and parameter estimation

### Descriptive results

Table 1 presents the proportion of the sample in each of the five states at each survey wave, broken down by sex and race/ethnicity. Table 2 presents the overall average transition probabilities between the five states across all waves for the full analytic sample. We present bar graphs that compare the percentages in Round 1 with those in Round 7 for each of the five compartments in the Supporting Information [S2–S6 Figs: S1 File].

**Table 2:** Average wave-to-wave transitions for full NLSY-97 analytic sample.

	Wave *t* + 1					
Wave *t*	X	C1	A	C2	R	Total
X	0.902	0.070	0.026	NA	NA	1.00
C1	0.582	0.323	0.096	NA	NA	1.00
A	NA	NA	0.286	0.221	0.508	1.00
C2	NA	NA	0.2	0.346	0.455	1.00
R	0.288	NA	0.108	0.127	0.477	1.00

At any given survey wave, most sample members are in state *X* (those not yet criminally active or arrested and those rehabilitated), but this share drops over time (from 83% at Round 1 to 74% at Round 7). A greater proportion of women than men are in state *X* at each survey wave, and this sex gap widens over time, as more men than women leave state *X* because they begin offending or are arrested. The flow of people out of state *X* is especially high among Black men, 79% of whom are in *X* at Round 1 compared to only 66% at Round 7.

The share of people in *C*_1_ (criminally active without an arrest) doubles between Rounds 1 (7%) and 2 (14%) but declines over subsequent survey waves, reaching a low of 4% at Round 7. More men than women are in *C*_1_ at any given wave, but the sex gap narrows over time, and there are relatively small differences by race/ethnicity.

The overall share of people in state *A* (recently arrested) remains fairly stable over time, but there is considerable variation by sex and race/ethnicity. Especially noteworthy is the large share of Black and Hispanic men among those who have been recently arrested compared to White men. At Round 1, roughly 7% of Black and Hispanic men are in state *A* compared to 4% of white men, and these gaps widen over time, such that by Round 7, 10% of Black men and 8% of Hispanic men in state *A* compared to 5% of White men.

There are fewer people in state *C*_2_ (those currently criminally active with a history of arrest) than any other state at any given survey wave. More men than women are in state *C*_2_ at all time periods, and the sex gap increases over time.

The share of people in state *R* (previously arrested but without a record of offending or arrest in the past year) starts off very low in early survey waves but increases over time. A sex gap also emerges at later survey waves, with more men than women represented in *R*. This is especially noticeable among Blacks by Round 7, during which 15% of Black men are in *R* compared to only 5% of Black women.

Table 2 shows the population “flows” between states from one wave to the next. First, consider flows from state *X*. Most people (90%) who are in state *X* at a given wave remain in *X* at the next wave. The biggest flow out of *X* is into state *C*_1_ (7%), onset of offending without arrest. A smaller group of people (3%) who start in *X* are arrested at the next wave (state *A*).

Most people (58.2%) in state *C*_1_ at a given wave return to *X*, desisting from criminal activity, while roughly a third remain in *C*_1_. A smaller share (10%) report an arrest at the next wave, moving to *A*.

About half of the people in state *A* at a given wave move to *R* at the subsequent wave, which means that by that time they had not offended or been arrested in the past year. A little more than a quarter (29%) of those in state *A* at a given wave remain there (are rearrested), while 22% move to state *C*_2_, remaining criminally active but without another arrest.

Examining transitions from state *C*_2_, we find that the most common transition (46%) is to state *R* (desistance from offending); 35% of those in *C*_2_ at a given wave remain there in the subsequent wave, while 20% move to state *A*.

Finally, for those in state *R* at a given wave (arrested during the study period but have neither offended nor been arrested during the past year), over three-quarters remain criminally inactive and arrest-free by the subsequent wave, either because they remain in state *R* (48%) or transition back to *X* (29%); 13% of those in *R* transition to *C*_2_ and 11% transit back to *A*.

### Statistical estimation of transition rates by race and sex

**Fig 13:**
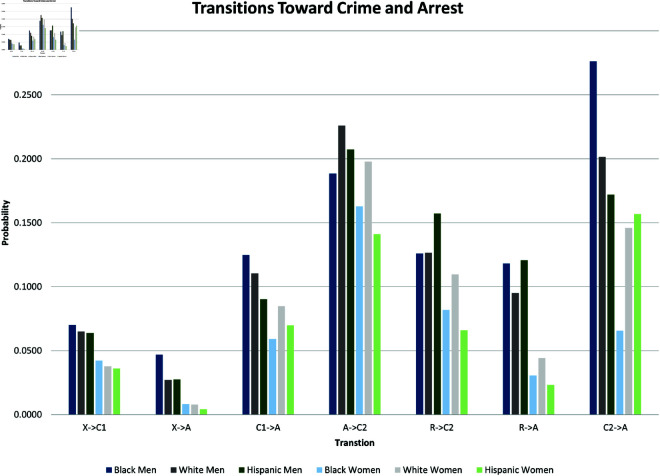
Transitions toward crime and arrest.

**Fig 14:**
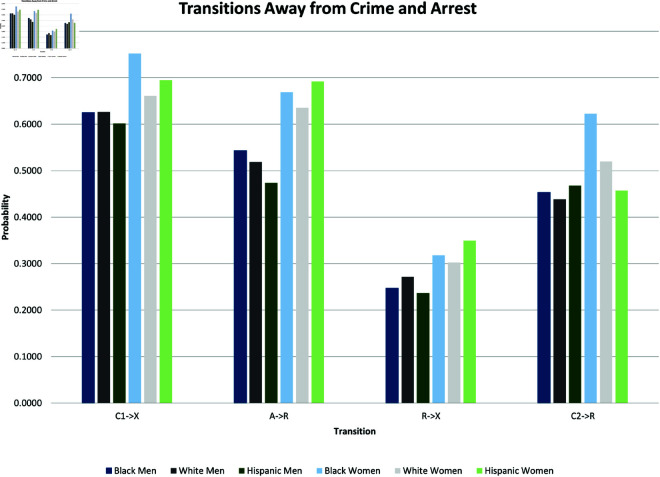
Transitions away from crime and arrest.

In the next stage of our analysis, we analyze differences across subgroups defined by sex and race/ethnicity in each of the transitions just described. We estimate the random effects logistic regression presented in Table 3. We then present the results graphically, by plotting predicted probabilities of each transition from the random effects logistic regression for each subgroup. Subgroup differences in the predicted probabilities for each of the eleven transitions are shown in Figs 2–12. Their means are presented as bar graphs in Figs 13 and 14.

**Table 3:** Estimated coefficients from random effects regression.

	$X\to C_{1}$	X→A	$C_{1}\to X$	$C_{1}\to A$	$A\to C_{2}|$	A→R	$C_{2}\to A$	$C_{2}\to R$	$R\to C_{2}$	R→A	R→X
Age	-0.29**	-3.60**	0.11	-0.11**	-0.16***	0.23***	-0.05	0.04	-0.27***	-0.05	0.24***
	(0.02)	(0.02)	(0.03)	(0.04)	(0.03)	(0.03)	(0.04)	(0.04)	(0.05)	(0.05)	(0.04)
Age	-0.02	-0.02**	0.02	-0.04**	0.00	0.00	0.01	-0.02	0.05**	0.01	-0.05***
Squared	(0.00)	(0.02)	(0.01)	(0.01)	(0.01)	(0.01)	(0.02)	(0.02)	(0.02)	(0.02)	(0.02)
Female	-0.57	-0.99	0.21	-0.36*	-0.14	0.51**	-0.51 +	0.43 +	0.06	-0.70**	0.12
	(0.09)	(0.13)	(0.13)	(0.16)	(0.18)	(0.17)	(0.28)	(0.24)	(0.26)	(0.29)	(0.15)
Black	0.11	0.56***	0.01	0.13	-0.22	0.08	0.41 +	0.07	-0.02	0.24	-0.11
	(0.11)	(0.12)	(0.14)	(0.16)	(0.16)	(0.15)	(0.22)	(0.23)	(0.25)	(0.23)	(0.15)
Hispanic	0.01	0.01	-0.12	-0.23	-0.10	-0.23	-0.17	0.11	0.30	0.26	-0.18
	(0.12)	(0.14)	(0.15)	(0.19)	(0.18)	(0.18)	(0.26)	(0.24)	(0.28)	(0.27)	(0.17)
Female*	-0.52	-0.52	0.45	-0.5	-0.05	0.13	-1.37**	0.47	-0.44	-0.64	0.28
Black	(0.15)	(0.21)	(0.21)	(0.22)	(0.32)	(0.3)	(0.63)	(0.45)	(0.45)	(0.51)	(0.25)
Female*	-0.06	-0.53	0.27	0.05	-0.35	0.59 +	0.29	-0.35	-0.95	-0.87	0.49 +
Hispanic	(0.17)	(0.25)	(0.25)	(0.24)	(0.39)	(0.36)	(0.53)	(0.47)	(0.53)	(0.62)	(0.38)
Constant	-3.10***	-3.70***	0.48***	-1.97***	-1.28***	0.09	-1.40***	-0.2	-0.247***	-2.39***	-0.91***
	(0.07)	(0.14)	(0.10)	(0.052)	(0.12)	(0.10)	(0.17)	(0.15)	(0.24)	(0.23)	(0.10)

The graphs in Figs 2 and 3 show the predicted transition probabilities for those who had not yet reported any criminal behavior or arrests. Women were more likely than men to remain in *X* at the subsequent wave and less likely to transition to states *C*_1_ or *A*. Whites were more likely than Blacks to remain in state *X* from one wave to the next and less likely to move from *X* to *A*, meaning that they had a lower risk of arrest. The probability of transiting from *X* to *A* was significantly higher for Black men than any other group and was lowest among Hispanic women. There were also significant interactions between sex and race/ethnicity in the X→A transition; the gap between men and women was larger among Blacks and Hispanics compared to Whites.

The graphs in Figs 4 and 5 show transition probabilities from state *C*_1_ at a given wave, but there were no significant differences by sex or race in the probability of remaining in *C*_1_ from one wave to the next. There was a significant interaction between sex and race in predicting the probability of moving from *C*_1_ to *X*, away from criminal behavior; Black women were especially likely to experience this transition. There was also a marginally significant (*p*<.10) interaction between race and sex in the transition from *C*_1_ to *A*. Black men were significantly more likely than Black women to make this transition; the sex gap was smaller among Whites and Hispanics.

Figs 6 and 7 focus on transitions from state *A*, those recently arrested. There were no significant differences by sex or race/ethnicity in the probability of moving from *A* to *C*_2_. The probability of transitioning from *A* to *R* (desistance from criminal activity by the recently arrested) was higher for women than for men, and it was especially high among Hispanic women (the interaction term between female and Hispanic was marginally significant, *p*<.10). Not shown in these figures are the findings that men were more likely than women to remain in A (rearrest) at the subsequent wave, and that Hispanic men were more likely than White men to remain in A.

The graphs in Figs 8 and 9 show subgroup differences in the transition probabilities for people in state *C*_2_ at a given wave. Blacks were less likely than Whites to remain in *C*_2_ from one wave to the next. Although the gap between Blacks and Whites in the predicted probabilities for the transition from *C*_2_ to *C*_2_ was larger among men (0.257 for Blacks compared to 0.343 for Whites) than women (0.305 for Blacks compared to 0.330 for Whites), there was no significant interaction between being Black and female. Black men in *C*_2_ were more likely to move to state *A*, (rearrested by the subsequent wave) compared to either White men (*p*<.07) or Black women (*p*<.03). There were no significant differences across racial/ethnic groups in the transition from *C*_2_ to *R*. Women in *C*_2_ were marginally more likely (*p*<.08) than men to desist and transit from *C*_2_ to *R* at the subsequent wave.

The graphs in Figs 10, 11, and 12 show subgroup differences in the transition probabilities for those in state *R* at a given wave. There were no significant differences by sex or race/ethnicity in the likelihood of remaining in *R* at the subsequent wave or transitioning to *C*_2_. Men were more likely than women to transition from *R* to *A*, (criminal activity and rearrest after a year without either), but there were no differences across racial/ethnic groups in this transition. Women were more likely than men to make the transition from *R* to *X* (three years without offense or arrest), but the sex gap was only significant only among Hispanics.

We summarize the main findings with regard to differences by sex and race/ethnicity presented in Figs 2–12.

- The likelihood of remaining in state *X*, without onset of criminal behavior or arrest, was significantly lower for men (compared to women) and Blacks (compared to Whites).
- Men were more likely than women to experience all transitions to *A*, in that they had a higher risk of arrest or rearrest.
- There were race differences in some transitions leading to *A*. Most notably, Black men were at a significantly higher risk of transitioning from X→A.
- In contrast, there were no racial/ethnic differences in the $X\to C_{1}$ transition, the onset of criminal offending without an arrest.
- Black women were more likely than either White women or Black men to transit from $C_{1}\to X$.
- Hispanic women were more likely (compared to Hispanic men) to transition from A→R and R→X.
- Hispanic women were less likely than all other groups to transit from $X\to C_{1}$.

The bar graphs in Figs 13 and 14 summarize the means of the distributions presented in Figs 2–12.

**Fig 15:**
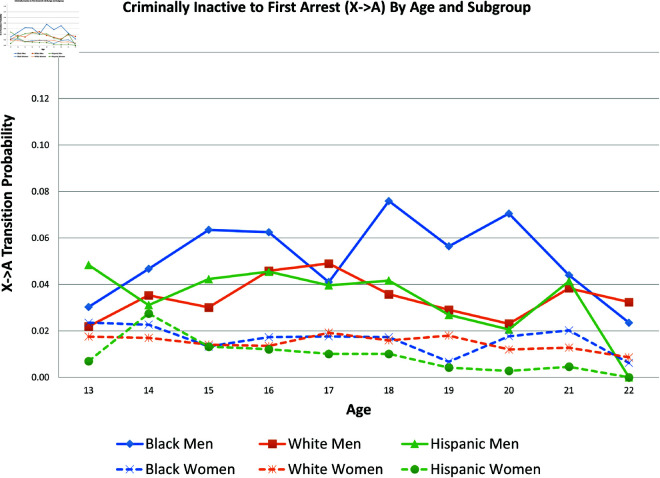
X→A by age and subgroup.

**Fig 16:**
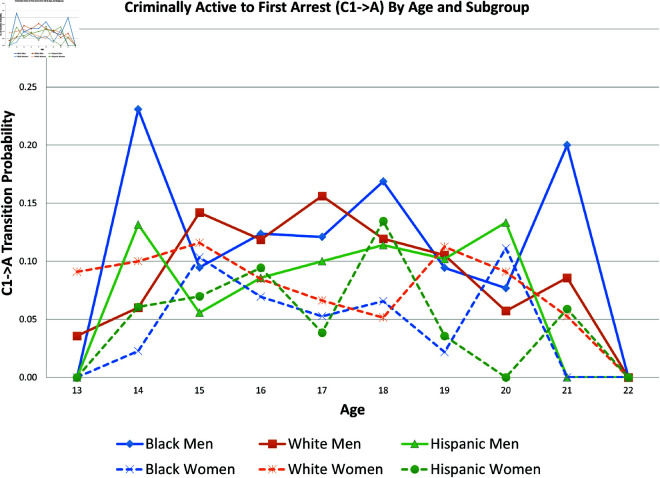
$C_{1}\to A$ by age and subgroup.

Fig 15 presents the probability of the X→A transition *by age* for each of the six subpopulations; because of sample size concerns, it uses the raw data, not the statistical estimations. Fig 16 uses the raw data to present the probability of the $C_{1}\to A$ transition *by age* for each of the six subpopulations.

### Parameter estimation

Earlier, we cited a number of dynamical systems studies of the spread of crime. Many of these assigned numerical values to the transition rates in their model to gain insights and to suggest policy. Their parameter values came from different sources. Different papers use different parameter values for the same transition. For example, Satish *et al*. [73] parameterize the rate at which those released from jail avoid further criminal activity as 0.088 (‘’recovery rate”), while Aguadze *et al*. [35] parameterize that rate as 0.8. (Our estimate for $A\to C_{2}$ is 0.58.) A primary goal of many systems modeling papers is to use a carefully constructed model and relevant data to estimate the important transmission parameters in the phenomenon under study. This is especially true for estimating contagion in the study of disease spread; see, for example, [74–77]. We have worked to combine our dynamical system model with a carefully constructed data set to estimate key transition rates in the spread of crimes. We consider these estimates, as summarized in Table 2 and Figs 13 and 14, a major contribution of our paper and hope that other crime researchers will find them useful.

### Convergence to equilibrium

For each of the six subpopulations, and then for the total population, we substitute the estimated values of the transition parameters for that subpopulation into dynamical system (2) in the text. Then, for each subpopulation, we use the Round 1 survey values to set the initial values $X(0),C_{1}(0),A(0),C_{2}(0),R(0)$ of each of the five states. We simulated system (2) to calculate the corresponding values at *t* = 1 from the values at *t* = 0, and so on for t=2,3,4,…. This process converges to a unique “long run" equilibrium in each state whose values are independent of choice of initial conditions. Expression (4) in the text provides a formula for this equilibrium X^*^ for state *X* for any choice of the transition parameters. Table 4 summarizes the convergence from initial conditions (column 2 in the table) to equilibrium distribution (column 6). Columns 3,4, and 5 illustrate how quickly this convergence occurs. We present bar graphs of the simulated long run equilibrium by subgroup in the Supporting information [S7 Fig: S1 File].

**Table 4:** Dynamics to equilibrium for Black Men and for White Men.

Black Men	Initial	6 Steps	10 Steps	50 Steps	Equilibrium (300 steps)
X	0.794	0.679	0.657	0.642	0.642
C1	0.097	0.065	0.062	0.060	0.060
A	0.070	0.089	0.093	0.095	0.095
R	0.016	0.124	0.142	0.152	0.152
C2	0.024	0.041	0.047	0.051	0.050
White Men	Initial	6 Steps	10 Steps	50 Steps	Equilibrium (300 steps)
X	0.830	0.761	0.745	0.734	0.734
C1	0.096	0.068	0.066	0.065	0.065
A	0.044	0.056	0.059	0.060	0.060
R	0.016	0.081	0.092	0.100	0.100
C2	0.015	0.033	0.038	0.041	0.041

### Results of the sensitivity simulation

#### Small changes.

To further examine the sensitivity of the equilibrium state distribution to the underlying parameters, we empirically computed and verified the theoretical expressions for the partial derivatives in the section on the Effect of Parameter Changes on X^*^. For Black and White males, we increased the decimal value of each parameter by 0.01 and computed the simulated change in X^*^. We found in all cases that this marginal impact matched the theoretical partial derivative calculations.

If a partial derivative of X^*^ for some transition parameter is close to zero, then the estimated value of X^*^ will not change much if slightly inaccurate values of that parameter are reported. Additionally, a marginal independent increase in such a transition parameter will have little impact. On the other hand, a partial derivative with a large magnitude indicates both a parameter that requires a more careful estimate from the data, and a potentially sensitive intervention target that could have a large impact on the long-run level of crime.

**Fig 17:**
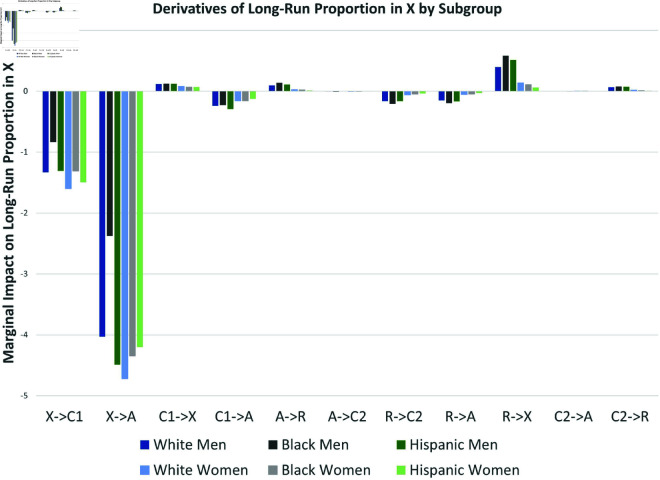
Derivatives of equilibrium proportion in X.

The partial derivatives are illustrated in Fig 17. As expected, increasing transition probabilities from *X* into crime or arrest $\alpha_{x1}$, $\alpha_{rc}$, $\alpha_{xa}$, $\alpha_{ra}$ decreased the long-run proportion X^*^ of law-abiding citizens; increasing desistance rates $\beta_{cx},\beta_{ar},$ from criminal behavior, and increasing rehabilitation $\epsilon_{rx}$ increased X^*^. Interestingly, changes in $\gamma_{c2}$ have negligible effects, while an increase in $\gamma_{c1}$ appears to decrease X^*^ (increase crime). In principle, the effects of these parameters are ambiguous, but it happens to be the case that, given the other parameter estimates from the data, marginal independent increases in the arrest of first-time offenders increases crime for all subgroups.

**Fig 18:**
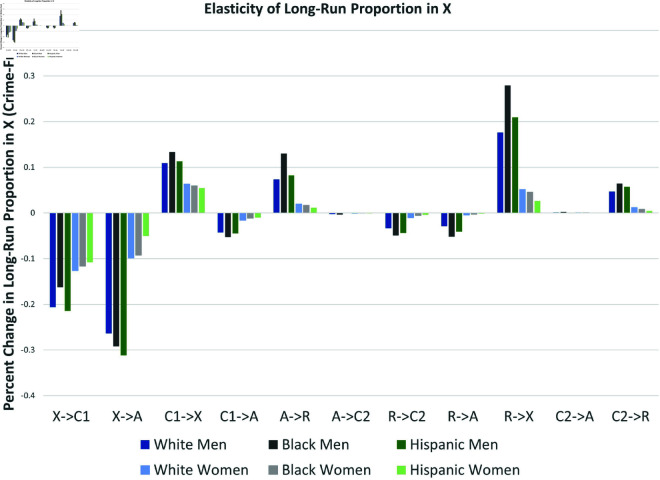
Elasticities of equilibrium proportion in X.

X^*^ could be more sensitive to percentage point changes in some parameters than others simply because their current estimated values are closer to zero. To account for this, and given the wide range of values in Table 2, it may be helpful to use percent changes rather than percentage point changes—that is, with elasticities. Fig 18 presents the percent change in the equilibrium value X^*^ given a one-percent change in each transition rate.

Overall, the long-run proportion of law-abiding citizens X^*^ is generally most sensitive to changes in the initial transitions into criminal activity (X→A and $X\to C_{1}$). The rehabilitation parameter R→X appears to be almost as important as the $X\to C_{1}$ for White and Hispanic men, and even more important for Black men. For men, pre-arrest desistance $C_{1}\to X$ is at least as important as post-arrest desistance (A→R, $C_{2}\to R$). Male crime levels are more sensitive to post-arrest transitions than female crime levels, especially for Black males. Finally, note that preventing flows into crime and arrest for never-offenders (e.g., decreasing X→A and $X\to C_{1}$) has more impact than preventing recidivism and arrest for inactive past offenders (e.g., decreasing $R\to C_{2}$ or R→A). For women, focusing percent changes on “pre-arrest” parameters is most effective with no exceptions.

Our mathematical analysis in the Section on Parameter Changes indicated that increases in the arrest parameters $\gamma_{1a}$ ($C_{1}\to A$) and $\gamma_{2a}$ ($C_{2}\to A$) have ambiguous effects on X^*^. Essentially, if criminals with no arrest history are unlikely to desist without arrest, higher arrest rates lead to lower long run crime levels ($\partial X^{*}/\partial \gamma >0$). Otherwise, higher arrest rates can increase the equilibrium level of crime—essentially by elongating the dynamic process of their return to long term law-abiding behavior. Using the NLSY97 data we estimated this effect for all subgroups. As indicated in Fig 17, for all six subgroups in our sample, $\partial X^{*}/\partial \gamma_{1a}$ is negative; a marginal increase in arrest rates increases long-run crime for all criminally active subgroups with no arrest history. On the other hand, $\partial X^{*}/\partial \gamma_{2a}$ is essentially negligible; increasing arrest rates of recidivists has basically no effect on long-run crime levels. Decreasing the virgin arrest rate for the criminally active would have a larger impact on male crime than female crime levels, and Black males most of all.

#### Large changes.

Many proposed interventions involve large changes in some parameter, e.g., closing prisons or decriminalizing certain drugs. Moreover, the effects of changes in each parameter may depend on its current value. In this Section we investigate the sensitivity of X^*^ to large changes in each parameter. To investigate the possible effects of larger changes for each subpopulation, we vary each transition parameter one at a time over a wide range that still ensures all probabilities are between zero and one.

**Fig 19:**
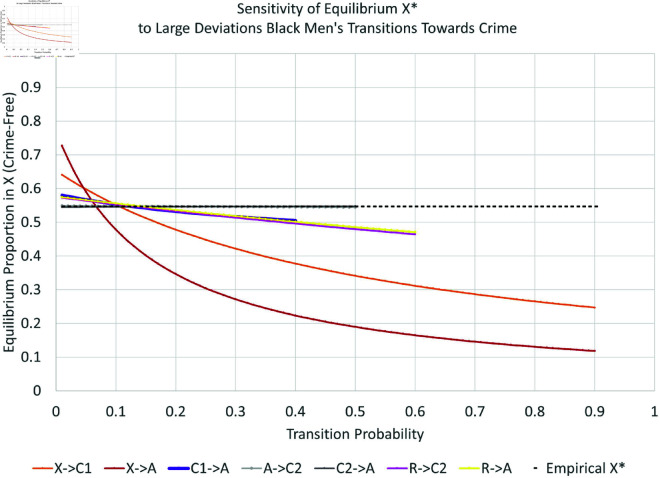
Large changes in transitions towards crime: black men.

**Fig 20:**
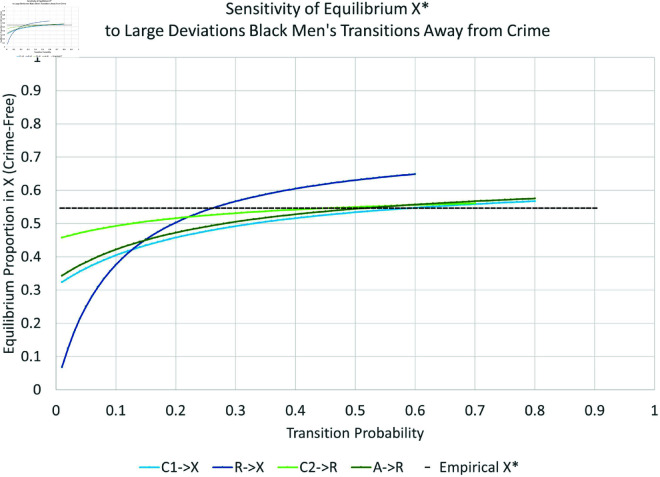
Large changes in transitions away from crime: black men.

**Fig 21:**
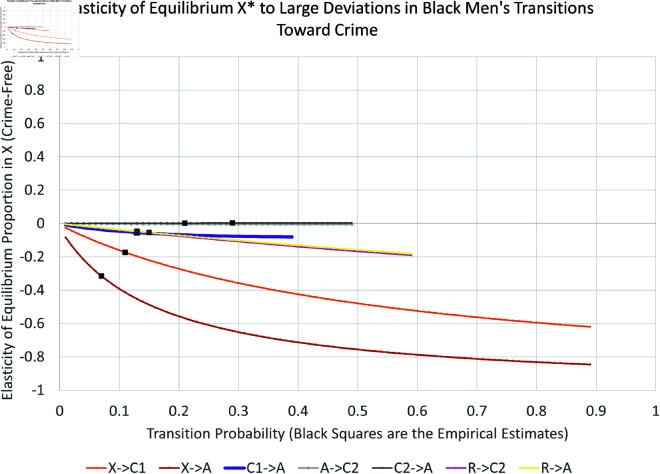
Elasticity of large changes in transitions towards crime: black men.

**Fig 22:**
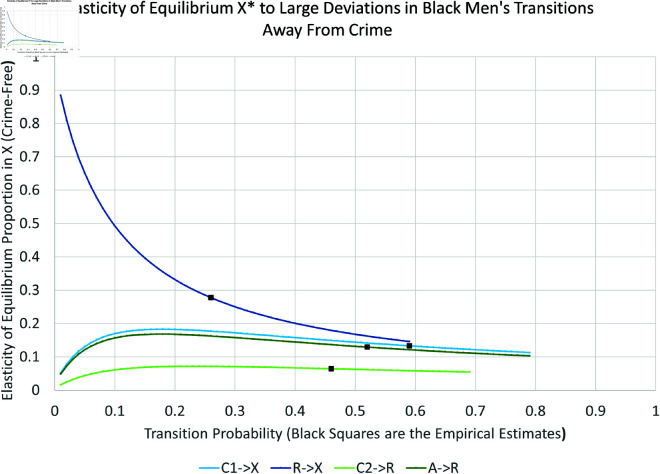
Elasticity of large changes in transitions away from crime: black men.

We present the simulation for Black males in Figs 19, 20, 21, and 22. The graphs for all six subpopulations are located in the Supporting Information [S8–S19 Figs: S1 File]. In Figs 19 and 20, the solid black line indicates the equilibrium X^*^. Increasing curves indicate an increase in law-abiders (and therefore, a decrease in law-breakers); the opposite holds for decreasing curves. For each parameter curve in these figures, the estimated value of the underlying parameter lies at its intersection with the solid black line. The slopes of the curves at these points represent the marginal impact of one percentage point increase in a parameter on the equilibrium proportion of people in *X*. They are consistent with the partial derivative calculations in the previous Section.

As one might expect, the biggest impacts on the level of crime-free X^*^ arise from changes in the rates for leaving X^*^ via new criminal activity—both with arrest ($\alpha_{xa}:X\to A$, bottom curve in Fig 19) and without arrest ($\alpha_{x1}:X\to C_{1}$, second lowest curve in Fig 19). For example, changing $\alpha_{xa}$ can drop the equilibrium percentage of crime-free Black males 43 percentage points from its current equilibrium at 55%, or raise it by 18 percentage points. Similar changes for Black females can decrease X^*^ from 82% to 16%. Increases in $\gamma_{1a}:C_{1}\to A$ increase crime to a smaller degree. Increases in $\gamma_{2a}:C_{2}\to A$ have no impact on the equilibrium crime rate, as indicated by its nearly horizontal curve. Increasing the arrest rate for those with an arrest history has no effect in contrast to the effect of arresting those with no arrest history. This turns out to be true for all six subgroups. The rehabilitation parameter R→X is the third most-important transition for long-run Black male crime levels from the perspective of percentage-point changes.

Finally, Figs 21 and 22 indicate the *percent* changes in the crime-free equilibrium X^*^ in response to *percent* changes in the transition probabilities—the elasticity approach. The black dots in these plots indicate the empirically estimated levels. The equilibrium is most sensitive to percent changes in X→A and R→X for Black men. The X→A response could become even more elastic for larger values of X→A, while the R→X response would become even more elastic for smaller values of R→X.

## Results on racial differences via simulations

In the previous Sections we examined the impacts of independent changes in the transition parameters of our model on the equilibrium level of crime in order to discover which paths might most efficiently lead to decreases in the level of crime. In this Section we use our model to shed light on which transitions contribute most to differences in the equilibrium levels of crime for Black and White males. We focus on Black and White males because the equilibrium X^*^ is quite similar for Black and White females.

Using the transition probabilities estimated for Black and White men in Figs 2–12, we calculate the absolute and relative Black-White male differences for each parameter. We then examine how decreases in these differences would affect the differences in the equilibrium X^*^ for Black and White males.

### Percent differences in transition probabilities

The last two rows in Table 5 present the absolute and relative differences between Black and White males for each of the eleven transition parameters in our model. We use


$$\frac{\text{White rate}-\text{Black rate}}{\text{Black rate}}.$$

to express the relative differences.

**Table 5:** Black-white male probability differences.

		$X\to C_{1}$	X→A	$C_{1}\to X$	$C_{1}\to A$	$A\to C_{2}$	A→R	$R\to C_{2}$	R→A	R→X	$C_{2}\to A$	$C_{2}\to R$
Men	Probability	0.070	0.047	0.625	0.125	0.188	0.543	0.126	0.118	0.247	0.276	0.453
	Standard Error	0.074	0.026	0.132	0.007	0.064	0.171	0.108	0.055	0.104	0.058	0.123
Men	Probability	0.065	0.027	0.627	0.111	0.226	0.519	0.126	0.095	0.272	0.201	0.439
	Standard Error	0.072	0.015	0.137	0.007	0.073	0.171	0.110	0.043	0.111	0.055	0.122
	Absolute Difference	0.005	0.020	-0.001	0.014	-0.038	0.025	0.000	0.023	-0.025	0.075	0.015
	Relative Difference	0.078	0.718	-0.002	0.130	-0.166	0.047	-0.004	0.244	-0.090	0.371	0.034

As the next-to-last line in Table 5 indicates, the largest absolute difference is the 7.5 percentage point difference in the $\gamma_{2a}:C_{2}\to A$ parameter between the Black rearrest probability of 27.6% and the White rearrest probability of 20.1%. The second largest absolute difference was the 3.8% advantage for White male recidivists avoiding rearrest $\zeta_{a2}:A\to C_{2}$.

Reading the bottom line in Table 5, we see that the largest relative difference was the 72% difference in the arrest rate for new offenders $\alpha_{xa}:X\to A$. In this list of relative differences, the probability of re-arrest after returning to crime ($\gamma_{2a}:C_{2}\to A$), which had the largest *absolute* difference, had the second largest *relative* difference.

### Systemic effects

**Fig 23:**
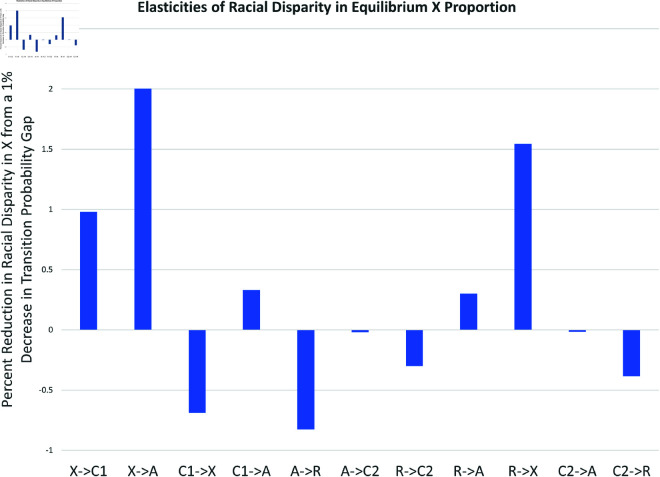
Elasticity of black-white male disparity in X.

Next, we examine the impact that these absolute and relative differences had on the equilibrium values of X^*^ for Black and White males. First, we ran our simulations with a one percent reduction in the absolute difference for any fixed transition probability. Fig 23 shows the percent reduction in the Black-White equilibrium value of X^*^ given this 1% reduction in any transition parameter gap. Once again, the early arrest transition $\alpha_{xa}:X\to A$ had the highest impact; a one percent reduction in the gap between the Black $\alpha_{xa}$ and the White $\alpha_{xa}$ reduced the X^*^ racial difference by 2%. The next highest impacts came from reducing the R→X gap for a reduction in the X^*^ difference of 1.5% and reducing the $X\to C_{1}$ gap for an equilibrium reduction of 1%. Reducing the $C_{2}\to A$ gap had essentially no effect on the X^*^ difference, but reducing the $C_{1}\to A$ gap reduced the X^*^ racial difference by about 0.3%.

**Fig 24:**
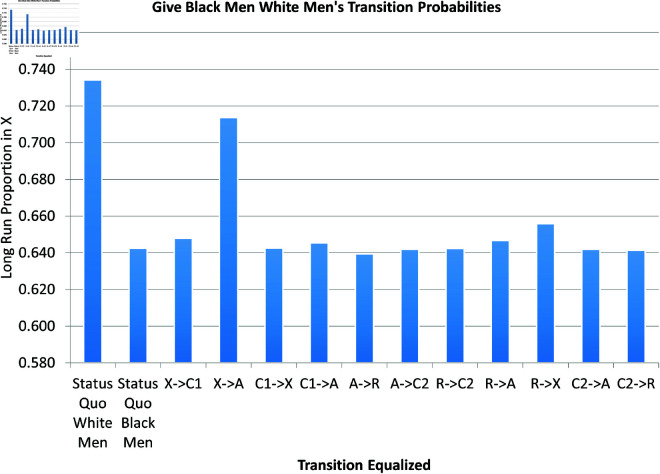
Black-white male disparity in X when equalizing each transition.

Finally, instead of a 1% reduction in the gap between transition probabilities, we examined the result of closing the gap. More precisely, we ran our dynamic simulation (1) with the transition parameters for Black males with one exception; we changed one parameter to the corresponding value for White males. We repeated this for each parameter. The results are pictured in Fig 24. The actual sizes of the gaps in the transitions matters in Fig 24. The X→A transition dramatically constituted the largest percent gap; the impact of closing this gap dwarfs the impact of closing any of the other gaps. The R→X parameter comes in a distant second place.

When we changed one of the White men’s transition probabilities to that of Black men, the same general conclusions held. In all cases, the racial equalization of the X→A transition dwarfed the equalization of the other transitions in terms of the impact of the racial gap in total crime and X. This is illustrated in the Supporting information in S20 and S21 Figs [S20–S21 Figs: S1 File].

## Discussion

This paper combines an analytic model with a rich data set to gain policy-relevant insights on the dynamics of criminal involvement. Our model has led us to focus on the eleven transition parameters in Fig 1. We have noted many strong sex-based differences in yearly transitions into and out of self-reported crime and arrest, and a few strong racial differences. The rate at which criminally active Black women with no arrest history desisted from crime in the next year ($\beta_{1x}:C_{1}\to X$) was substantially larger than the rate for White women and represented the largest racial difference for women. Other strong racial differences appeared in yearly transitions into arrest. We have also studied the systemic effects of small and large changes in the parameters on long run crime levels for each subgroup, and the impact of closing racial differences in the parameters on racial differences in long run crime levels. While our conclusions are not prescriptive of specific policy interventions, they are informative for ascertaining the interactive effects of common targets of intervention on long run crime and group differences therein. We summarize these insights below.

The $\gamma_{2a}:C_{2}\to A$ transition parameter (arrest rate of criminals with an arrest history) differed the most between White men and Black men. However, increasing $\gamma_{2a}:C_{2}\to A$ had a negligible impact on long-run crime. Increasing the $\gamma_{1a}:C_{1}\to A$ transition parameter (arrest rate of criminals with no arrest history) had a larger impact, increasing long-run crime for all subgroups. *Equalizing* the $\gamma_{1a}:C_{1}\to A$ transition parameter across races had a larger impact on reducing racial differences in long run crime than the $\gamma_{2a}:C_{2}\to A$ transition parameter. In theory, the effect of increases in each parameter is ambiguous and depends on other transition rates, such as the likelihood of desistance among those with and without an arrest history. But at the estimated parameter values for all subgroups, marginal, independent decreases in virgin arrests (but not repeat-arrests) will decrease long run crime. This is in part due to relatively high desistance rates among criminally active people with no arrest history (e.g., nearly 60% of Black men with no arrest history tend to desist in the next year without arrest). In contrast, the first arrest exposes individuals to higher future offense and lower desistance rates. Consequently, reducing the Black-White gap in first-time arrests may help reduce the racial gap in long run crime between Black and White men, by protecting Black men from the criminogenic effect of arrest. This finding is informative for policies that affect arrest rates, since it shows that aggressively arresting Black men without an arrest history can actually increase long run crime and racial differences in long run crime. More generally, it shows that pre-arrest desistance rates are sufficiently high for all subgroups such that increases in first-time arrests may be criminogenic in the long run regardless of race or sex.

With one exception, the $\alpha_{x1}:X\to C_{1}$ (“onset" without arrest) probability was generally the second most important transition for all subgroups in terms of the local sensitivity of the long-run crime level, in line with the mantra “prevention is the best cure." The exception was Black men. While $\alpha_{x1}:X\to C_{1}$ was still important for Black men, their long run crime outcomes appear to be even more sensitive to changes in the rehabilitation ($\epsilon_{rx}:R\to X$) rate. Consequently, equalizing the rehabilitation parameter appears to have the second largest effect on the racial differences in long run crime outcomes between Black and White men, highlighting the importance of supporting criminally inactive Black men with an arrest history.

The $\alpha_{xa}:X\to A$ transition parameter (rate of first arrest among law-abiding citizens with no arrest history) had the largest *marginal* effect in reducing both crime levels and racial differences in crime level. However, we cannot state whether this is primarily due to actual racial differences in initial crime participation (which could reflect differences in neighborhood context, socioeconomics and educational opportunity), to racial differences in the policing of citizens with no arrest history, or both. Our analysis does suggest that this is a key leverage point for policy in the reduction in crime and in racial disparities in crime outcomes.

These questions have not, and could not have been answered with “reduced form," regression-based approaches alone. It is precisely because the effects of the “onset" rate into crime, arrest rates, desistance, rehabilitation, and recidivism are interdependent that understanding their relative importance for long-run crime requires systems thinking. We are not aware of previous literature that has systematically compared the contribution of these important criminological parameters to long-run crime and intergroup disparities in crime. These are new and nuanced contributions to the literature, made possible with our systems approach.

### Simplifying assumptions and limitations

Models are necessarily simplifications of real-world phenomena. Systems modeling begins with strong simplifying assumptions in order to build intuition on how the model components interact. Such simplifying assumptions in ecology, economics, and epidemiology usually include homogeneous agents, random mixing, no adaptation, and a loose relationship to real data [74] . To derive a model more useful for policy considerations, systems modelers systematically relax these simplifications and add more complex agents and relationships. Through this process, one learns the role of these complexities in the overall system.

**Homogeneity of agents.** The agents in our first model [33] were completely homogeneous. We added age structure in our second paper [34]. In this paper, we add heterogeneity by race and sex (and estimated parameters using a robust data set). There are still important heterogeneities to add before we can strongly suggest policy implications. We intend to add these in future iterations of our model.

**Neighborhood effects.** Neighborhood characteristics and historical context play a major role in shaping crime outcomes across different racial groups [78–81]. For example, Sampson and Neil [81] “link early-life social conditions to racial disparities in arrest over the life course and in changing times.” Their paper includes an extensive review of neighborhood effects. There is some information about underlying neighborhoods in the NLSY97 data set. We plan to incorporate this information in our next iteration, realizing that including such heterogeneities will substantially increase the number of model components. The resulting reduction of the number of agents in each component will challenge the robustness of our statistical analysis.

**Linear transitions.** In our first two papers [33, 34] we included the dynamic that crime could spread through interactions between those engaged in criminal activity and those who are not. In this paper, we assumed that each agent’s entry into criminal activity is unrelated to other agents (linear transitions). Both of these dynamics play a role. We hope to examine the role of contagion vs linear flow in commencement of criminal activity in future work, but our NLSY97 data are not able to reliably identify contagion.

**Definition of crime.** A major simplifying assumption is that a wide range of crimes are categorized as a generic crime in this model. The definitions of these crimes are discussed in the Data section. The NLSY97 data set includes different kinds of crime, and we hope to understand how our results might vary by crime type in future work.

**Equilibrium and fixed parameters.** For simplicity and tractability, we assumed that the transition parameters are independent of state sizes and fixed over time; and we focused on the long-run equilibria for various demographic subgroups. Yet, we know that crime has decreased since the turn of the 21st century, and the seven-year window of the NLSY97 data may not constitute a long-run equilibrium. This is a standard concern in equilibrium-based analyses, but we plan to account for these issues in future work. Time-varying parameters would present challenges for both empirical estimation and tractability for the theoretical model, so we begin by estimating fixed transition probabilities based in part on their seven-year averages in our panel.

**Policy interventions.** Many real world interventions can affect multiple parameters in our model. Our focus has been on the systemic effects of marginal, independent changes in particular target pathways of intervention—not the interventions themselves. While our conclusions are not prescriptive with respect to policy interventions, they are informative for understanding the dynamic interplay between common targets of intervention. For example, increasing police presence may increase the first-time arrest rate $\gamma_{1A}$ and deter initiation into crime $\alpha_{x1}$. This paper doesn’t tell us whether to increase police presence, but it does suggest that police officers may increase crime for all subgroups if they have a small effect on initiation and a large effect on first-time arrest rates.

### Conclusion

As columnist/humorist Frank Tyger famously states, “Listening to both sides of a story will convince you that there is more to the story than both sides." Systems thinking is about studying the consequences of the fact that each aspect of crime so often studied in isolation, is in fact interconnected. This has important implications for understanding which aspects to target with policy to reduce racial disparities in crime and crime in general. It may help us avoid unintended consequences—such as increasing crime levels among all subgroups through the arrest of first time offenders—and can work as complement, not a substitute, for research methods that isolate the causal effects of particular interventions on specific outcomes. This paper contributes to a growing body of literature evidencing the utility of systems thinking for fighting America’s systemic problems.

## Supporting information

S1 File Contains all Supporting Information, exposition of the 3D and 5D systems, and additional descriptive analyses of the data. (PDF)

S2 File Supplementary figures. (ZIP)
