# Supplementary material for: A dynamical systems analysis of criminal behavior using national longitudinal survey of youth data
Source: PLoS One. 2025 Aug 8;20(8):e0324014. doi: 10.1371/journal.pone.0324014 (PMC12334011; doi:10.1371/journal.pone.0324014)
Supplement: S1 File — (PDF) [file pone.0324014.s001.pdf]

SUPPORTING INFORMATION:

A Dynamical Systems Analysis of Criminal Behavior Using National Longitudinal Survey of Youth Data

Simple Three-Variable Model

Model Structure

To aid the reader's understanding of the mathematical analysis of our five-dimensional model, we carry out the corresponding analysis for a simplified three-dimensional model where the analysis is more transparent. Figure S1 summarizes this 3D model. The three states are X, C, A:

X: Those who have neither committed crime nor been arrested in the last year.

C: Those who have committed a crime in the last year but were not arrested.

A: Those who have been arrested in the last year, with or without crime commission.

Figure S1: Flow Diagram for the 3D Model

For a person in state X (someone who has not committed a crime or been arrested in the past year) in a given time period, σ_{xx} represents the probability of remaining in X (remaining free of crime and arrest) during the next time period, α_{xc} represents the probability of moving into C (becoming criminally active without arrest), and α_{xa} represents the probability of moving into A (moving to criminal activity and being arrested for that activity in the next year).

For a person in state C (someone who has committed a crime in the past year but not been arrested) in a given time period, σ_{cc} represents the probability of remaining in C (continuing to commit crime) during the next time period, γ_{ca} is the probability of moving to state A (being arrested), and β_{cx} is the probability of moving to state X (not having committed a crime or been arrested in the past year).

For a person in state A (someone who has been arrested in the past year) in a given time period, σ_{aa} represents the probability of remaining in A (being arrested again) during the

next time period, ζ_{ac} is the probability of moving to state C (committing crimes without being arrested), and β_{ax} is the probability of moving to state X (not having committed a crime or been arrested in the past year).

We use the Greek letter α for flows from crime-free to crime (with or without arrest), β for flows back to a crime-free status, γ for arrest, and ζ for return to crime after arrest. In each of the above sentences, the conditional probabilities sum to 1, as illustrated in the following equation.

(S.1) $$\sigma_{xx} = 1 - \alpha_{xc} - \alpha_{xa}, \quad \sigma_{cc} = 1 - \gamma_{ca} - \beta_{cx}, \quad \sigma_{aa} = 1 - \zeta_{ac} - \beta_{ax}.$$

For simplicity, we did not include the σs in Fig. S1. The following linear (Markov) system of equations captures this dynamic:

(S.2a) $$X(t+1) = \sigma_{xx}X(t) + \beta_{cx}C(t) + \beta_{ax}A(t)$$

(S.2b) $$C(t+1) = \sigma_{cc}C(t) + \alpha_{xc}X(t) + \zeta_{ac}A(t)$$

(S.2c) $$A(t+1) = \sigma_{aa}A(t) + \gamma_{ca}C(t) + \alpha_{xa}X(t)$$

Using (S.1), rewrite system (S.2) as:

(S.3a) $$X(t+1) - X(t) = \beta_{cx}C(t) + \beta_{ax}A(t) - \alpha_{xc}X(t) - \alpha_{xa}X(t)$$

(S.3b) $$C(t+1) - C(t) = \alpha_{xc}X(t) + \zeta_{ac}A(t) - \beta_{cx}C(t) - \gamma_{ca}C(t)$$

(S.3c) $$A(t+1) - A(t) = \gamma_{ca}C(t) + \alpha_{xa}X(t) - \zeta_{ac}A(t) - \beta_{ax}A(t)$$

Since the right hand sides of system (S.3) sum to 0,

(S.4) $$X(t+1) + C(t+1) + A(t+1) = X(t) + C(t) + A(t),$$

for all times t. The population has constant size N. If we divide each term in linear system (S.2) or linear system (S.3) by N, we replace X, C, A by $X/N, C/N, A/N$ and the three

variables in these systems become population *fractions* instead of total numbers. We will make these changes, but without changing the notation, so that we regard the three variables as population fractions.

Since the equations sum to zero, there is a redundant equation. Since the variables sum to 1, if we know two of the variables, we know the third. This means that we can write system (S.3) as system of two equations in two unknowns; for example, as:

$$C(t+1) - C(t) = -(\alpha_{xc} + \beta_{cx} + \gamma_{ca})C + (\zeta_{ac} - \alpha_{xc})A + \alpha_{xc} \tag{S.5a}$$

$$A(t+1) - A(t) = (\gamma_{ca} - \alpha_{xa})C - (\alpha_{xa} + \zeta_{ac} + \beta_{ax})A + \alpha_{xa} \tag{S.5b}$$

Long Run Equilibrium: Analytic Expression

Because of (S.1), system (S.2) is a Markov system of equations, which implies that every solution of (S.2) converges over time to the same long-run equilibrium X^* in population frequency space, from any non-zero starting point. To find X^*, we set the right-hand sides of system (S.5) equal to zero, which we rewrite as:

$$\alpha_{xc} = (\alpha_{xc} + \beta_{cx} + \gamma_{ca})C - (\zeta_{ac} - \alpha_{xc})A \tag{S.6a}$$

$$\alpha_{xa} = -(\gamma_{ca} - \alpha_{xa})C + (\alpha_{xa} + \zeta_{ac} + \beta_{ax})A \tag{S.6b}$$

and then solve for C and A, using standard linear algebra techniques. We then use $X = 1 - C - A$ to solve for the equilibrium X^*:

$$X^* = \frac{\beta_{cx}\zeta_{ac} + \beta_{cx}\beta_{ax} + \gamma_{ca}\beta_{ax}}{\alpha_{xc}(\zeta_{ac} + \beta_{ax} + \gamma_{ca}) + \alpha_{xa}(\zeta_{ac} + \beta_{cx} + \gamma_{ca}) + \beta_{cx}\zeta_{ac} + \beta_{cx}\beta_{ax} + \beta_{ax}\gamma_{ca}} \tag{S.7}$$

To understand (S.7) better, write it as:

$$X^* = \frac{1}{1 + \dfrac{\alpha_{xc}(\zeta_{ac} + \beta_{ax} + \gamma_{ca}) + \alpha_{xa}(\zeta_{ac} + \beta_{cx} + \gamma_{ca})}{\beta_{cx}\beta_{ax} + \zeta_{ac}\beta_{cx} + \gamma_{ca}\beta_{ax}}}. \tag{S.8}$$

The fraction in the denominator of (S.8) is an **output-input ratio**. The α_{xc} and α_{xa} in the numerator of this fraction parameterize the two direct paths out of X. The terms in the denominator parameterize the four paths into X: β_{cx} directly from C to X, β_{ax} directly from A to X, $\zeta_{ac}\beta_{cx}$ from A to X via C, and $\gamma_{ca}\beta_{ax}$ from C to X via A.

Long Run Equilibrium: Dependence on Parameters

Understanding how the equilibrium X^* depends on the various parameters of the model is crucial to the systems approach. How does increasing the arrest rate γ or decreasing the rate of recidivism ζ affect the relative number of non-law-abiding citizens. Some parameters are easy to work with. For example, the rates α_{xc} and α_{xa} appear only in the denominator of expression (S.7). Increasing either α decreases X^*; the faster the flow into criminal activity, the higher the crime rate.

Understanding the effects associated with other parameters requires taking partial derivatives of (S.7). For example, taking the partial derivative of X^* with respect to β_{cx} (after applying the quotient rule of calculus with some algebraic simplification) shows how increasing probability of desistance (β_{cx}) increases the fraction of people in X^*, which means lowering the crime rate):

$$\text{(S.9)}\quad \frac{\partial X^*}{\partial \beta_{cx}} = \frac{[(\zeta_{ac}+\beta_{ax})\alpha_{xc}+\alpha_{xa}\zeta_{ac}](\zeta_{ac}+\beta_{ax}+\gamma_{ca})}{(\alpha_{xc}(\zeta_{ac}+\beta_{ax}+\gamma_{ca})+\alpha_{xa}(\zeta_{ac}+\beta_{cx}+\gamma_{ca})+\beta_{cx}\zeta_{ac}+\beta_{cx}\beta_{ax}+\beta_{ax}\gamma_{ca})^2} > 0.$$

A parallel computation shows that $\dfrac{\partial X^*}{\partial \beta_{ax}} > 0$.

The effect on X^* of increasing the arrest rate (γ_{ca}) is more nuanced, because the effect depends on the relative size of β_{ax} and β_{cx}: Once again, the quotient rule and some algebraic simplification yields:

$$\text{(S.10)}\quad \frac{\partial X^*}{\partial \gamma_{ca}} = \frac{(\beta_{ax}-\beta_{cx})(\alpha_{xc}\beta_{ax}+\alpha_{xc}\zeta_{ac}+\alpha_{xa}\zeta_{ac})}{(\alpha_{xc}(\zeta_{ac}+\beta_{ax}+\gamma_{ca})+\alpha_{xa}(\zeta_{ac}+\beta_{cx}+\gamma_{ca})+\beta_{cx}\zeta_{ac}+\beta_{cx}\beta_{ax}+\beta_{ax}\gamma_{ca})^2}.$$

If being arrested has a positive effect on desistance, $\beta_{ax} > \beta_{cx}$, then increasing the arrest rate decreases crime. However, if the probability moving from being arrested makes becoming crime-free less likely, $\beta_{ax} < \beta_{cx}$, then increasing the arrest rate can increase the crime level.

For each of the first partial derivatives we have computed, the second partial derivative has the opposite sign. Therefore, any independent increase in a transition probability has decreasing returns on X^*. For example, any positive effect on the crime level of reducing the transition rate α_{xc} from no-crime to crime has a smaller effect the larger the value of α_{xc}.

Finally, we compute that

$$\text{(S.11)} \qquad \frac{\partial X^*}{\partial \zeta_{ac}} = \frac{(\beta_{cx} - \beta_{ax})(\alpha_{xc}\gamma_{ca} + \beta_{cx}\alpha_{xa} + \gamma_{ca}\alpha_{xa})}{(\alpha_{xc}(\zeta_{ac} + \beta_{ax} + \gamma_{ca}) + \alpha_{xa}(\zeta_{ac} + \beta_{cx} + \gamma_{ca}) + \beta_{cx}\zeta_{ac} + \beta_{cx}\beta_{ax} + \beta_{ax}\gamma_{ca})^2}.$$

Expression (S.11) suggests that if desistance is more likely for those not recently arrested ($\beta_{cx} > \beta_{ax}$), then decreasing ζ_{ac} might not decrease the crime rate.

We next expand our model to consider recidivism more directly. Such an expansion is important because recidivism plays an important role in the spread of crime and needs to be considered in any model that tries to capture real world crime dynamics.

Computing the equilibrium of the 5D System (S.1)

With the assistance of Mathematica, we computed that the long run equilibrium of system (1) is:

$$\text{(S.12a)} \qquad X^* = \frac{\epsilon_{rx}(\beta_{1x} + \gamma_{1a})(\gamma_{2a}\beta_{ar} + \beta_{2x}(\beta_{ar} + \zeta_{a2}))}{D}$$

$$\text{(S.12b)} \qquad C_1^* = \frac{\alpha_{x1}\epsilon_{rx}(\gamma_{2a}\beta_{ar} + \beta_{2x}(\beta_{ar} + \zeta_{a2}))}{D}$$

$$\text{(S.12c)} \qquad A^* = \frac{(\alpha_{x1}\gamma_{1a} + (\beta_{1x} + \gamma_{1a})\alpha_{xa})(\beta_{2x}(\epsilon_{rx} + \alpha_{ra}) + \gamma_{2a}(\alpha_{x2} + \epsilon_{rx} + \alpha_{ra}))}{D}$$

$$\text{(S.12d)} \qquad R^* = \frac{(\alpha_{x1}\gamma_{1a} + (\beta_{1x} + \gamma_{1a})\alpha_{xa})(\gamma_{2a}\beta_{ar} + \beta_{2x}(\beta_{ar} + \zeta_{a2}))}{D}$$

$$\text{(S.12e)} \qquad C_2^* = \frac{(\alpha_{x1}\gamma_{1a} + (\beta_{1x} + \gamma_{1a})\alpha_{xa})((\epsilon_{rx} + \alpha_{ra})\zeta_{a2} + \alpha_{x2}(\beta_{ar} + \zeta_{a2}))}{D}$$

where the common denominator is:

$$D = \{\alpha_{x1}[\epsilon_{rx}\gamma_{1a}\gamma_{2a}+\epsilon_{rx}\gamma_{2a}\beta_{ar}+\gamma_{1a}\gamma_{2a}\beta_{ar}+\gamma_{1a}\gamma_{2a}\alpha_{ra}+\epsilon_{rx}\gamma_{1a}\zeta_{a2}+\gamma_{1a}\alpha_{ra}\zeta_{a2}+\alpha_{x2}\gamma_{1a}(\gamma_{2a}+\beta_{ar}+\zeta_{a2})$$

$$+\beta_{2x}(\epsilon_{rx}(\gamma_{1a}+\beta_{ar}+\zeta_{a2})+\gamma_{1a}(\beta_{ar}+\alpha_{ra}+\zeta_{a2}))]$$

$$+(\beta_{1x}+\gamma_{1a})[\epsilon_{rx}(\gamma_{2a}(\beta_{ar}+\alpha_{xa})+\alpha_{xa}\zeta_{a2})+\alpha_{xa}(\gamma_{2a}(\beta_{ar}+\alpha_{ra})+\alpha_{ra}\zeta_{a2}+\alpha_{x2}(\gamma_{2a}+\beta_{ar}+\zeta_{a2}))$$

$$+\beta_{2x}(\epsilon_{rx}(\beta_{ar}+\alpha_{xa}+\zeta_{a2})+\alpha_{xa}(\beta_{ar}+\alpha_{ra}+\zeta_{a2}))]\}$$

We claim:

$$\frac{\partial X^*}{\partial \beta_{1x}} > 0, \qquad \frac{\partial X^*}{\partial \beta_{2r}} > 0, \qquad \frac{\partial X^*}{\partial \beta_{ar}} > 0, \qquad \frac{\partial X^*}{\partial \epsilon_{rx}} > 0,$$

We will prove the first of these; the proofs of the rest are similar. Write $D = D_1 + D_2$, where D_1 is the expression in the first two lines of D and D_2 is the rest. Write M for the numerator of X^*. Then,

$$\frac{\partial D}{\partial \beta_{1x}} = \frac{D_2}{\beta_{1x}+\gamma_{1a}} \qquad \text{and} \qquad \frac{\partial M}{\partial \beta_{1x}} = \frac{M}{\beta_{1x}+\gamma_{1a}}.$$

Then,

$$\frac{\partial X^*}{\partial \beta_{1x}} = \frac{1}{D^2}\left[\frac{M}{\beta_{1x}+\gamma_{1a}}(D_1+D_2) - M\frac{D_2}{\beta_{1x}+\gamma_{1a}}\right] = M\frac{D_1}{\beta_{1x}+\gamma_{1a}} > 0.$$

As before, the effects of an increase in the arrest rates are quite a bit more subtle. For re-arrests, the derivative is:

$$\frac{\partial X^*}{\partial \gamma_{2a}} = \frac{\epsilon_{rx}(\beta_{1x}+\gamma_{1a})(\beta_{ar}-\beta_{2r})[\alpha_{x1}\gamma_{1a}+(\beta_{1x}+\gamma_{1a})\alpha_{xa}][(\epsilon_{rx}+\alpha_{ra})\zeta_{a2}+\alpha_{r2}(\beta_{ar}+\zeta_{a2})]}{D^2}$$

The sign of this derivative of X^* with respect to γ_{2a} depends on β_{2r} and β_{ar}. Specifically, the derivative is nonnegative if $\beta_{ar} \geq \beta_{2r}$ and negative otherwise. Simply put, arresting criminals with an arrest history reduces crime if they are more likely to desist after arrest than before arrest.

The derivative for first arrests is:

$$\frac{\partial X^*}{\partial \gamma_{1a}} = \frac{\alpha_{x1}\epsilon_{rx}(\gamma_{2a}\beta_{ar} + \beta_{2r}(\beta_{ar} + \zeta_{a2}))}{D^2} \times \Bigg[\epsilon_{rx}[\gamma_{2a}\beta_{ar} + \beta_{2r}(\beta_{ar} + \zeta_{a2})]$$

$$-\beta_{1x}[\gamma_{2a}(\epsilon_{rx} + \beta_{ar} + \alpha_{ra}) + \zeta_{a2}(\epsilon_{rx} + \alpha_{ra}) + \alpha_{r2}(\gamma_{2a} + \beta_{ar} + \zeta_{a2}) + \beta_{2r}(\epsilon_{rx} + \beta_{ar} + \alpha_{ra} + \zeta_{a2})] \Bigg].$$

Our Data Set

Individuals in our sample are classified into one of these mutually exclusive states at each survey wave but over time they may move in and out of different states. Our primary outcomes for the statistical analysis presented below are wave-to-wave transitions between different states in our model, as illustrated by the arrows in Fig. 1. We analyze the following transitions:

- $X \to X$: Someone with no arrest history, or with an arrest history but no crime/arrest in the last three years, who was not criminally active at a given wave nor the next wave,
- $X \to C_1$: Someone with no arrest history, or with an arrest history but no crime/arrest in the last three years, who was not criminally active at a given wave but reports involvement in criminal offending at the next wave;
- $X \to A$: Someone with no arrest history, or with an arrest history but no crime/arrest in the last three years, who was not criminally active at a given wave but is arrested at the next wave;
- $C_1 \to C_1$: Someone with no arrest history who was criminally active at a given wave and the next wave;
- $C_1 \to X$: Someone with no arrest history who was criminally active (without a recent arrest) at a given wave but is no longer criminally active at the next wave;
- $C_1 \to A$: Someone with no arrest history who was criminally active (without a recent arrest) at a given wave and is arrestedat the next wave;
- $A \to A$: Someone who was arrested at a given wave and the next wave;
- $A \to C_2$: Someone who was arrested at a given wave and reports involvement in criminal offending at the next wave without arrest;

- $A \to R$: Someone who was arrested at a given wave and has no arrest or reports of criminal behavior at the next wave;
- $C_2 \to C_2$: Someone with a recent arrest history who is criminally active without arrest at a given wave and the next wave;
- $C_2 \to A$: Someone with a recent arrest history who is criminally active without arrest at a given wave, and and is arrested at the next wave;
- $C_2 \to R$: Someone with a recent arrest history who is criminally active without arrest at a given wave, but is not criminally active and has no arrests at the next wave;
- $R \to R$: Someone who has a recent history of arrest in the last three years, and has not been criminally active or arrested at a given wave, nor the next wave;
- $R \to A$: Someone who has a recent history of arrest in the last three years, and has not been criminally active or arrested at a given wave, but is arrested at the next wave;
- $R \to C_2$: Someone who has a recent history of arrest in the last three years, and has not been criminally active or arrested at a given wave, but reports involvement in criminal offending without an arrest at the next wave;
- $R \to X$: Someone who has a recent history of arrest in the last three years, but has not been criminally active or arrested at a given wave and who, by the next wave, has gone three years without any reports of criminal activity or arrest.

Compartment Sizes

Table 1 in the main text presents the proportion of the sample in each of the five states at each survey wave, broken down by sex and race/ethnicity. Figs. S2 through S6 present bar graphs that compare the percentages in Round 1 with those in Round 7 for each of the five compartments.

,

Figure S2: 1997 and 2003 Distributions for X

Figure S3: 1997 and 2003 Distributions for C1

Figure S4: 1997 and 2003 Distributions for A

Figure S5: 1997 and 2003 Distributions for R

Figure S6: 1997 and 2003 Distributions for C2

Table S1: Significance of State-Year Trends by Subgroup

**Subgroup**	**X**	**C1**	**A**	**R**	**C2**
Black Men	-0.077*** (0.014)	-0.175*** (0.023)	0.050* (0.022)	0.289*** (0.025)	0.119** (0.035)
Hispanic Men	-0.022 (0.016)	-0.170*** (0.015)	-0.003 (0027)	0.271*** (0.031)	0.087* (0.038)
White Men	-0.061*** (0.010)	-0.144*** (0.016)	0.049** (0.018)	0.294*** (0.020)	0.132*** (0.026)
Black Women	0.032 † (0.018)	-0.171*** (0.026)	-0.039 (0.039)	0.212*** (0.036)	0.083 (0.059)
Hispanic Women	0.053** (0.021)	-0.154*** (0.029)	-0.040 (0.050)	0.147** (0.043)	0.031 (0.067)
White Women	-0.006 (0.013)	-0.131*** (0.019)	-0.009 (0.027)	0.231*** (0.026)	0.069 † (0.037)

Table S1 summarizes, by subgroup, the significance of time trends in the proportion of people in each compartment. It reports the coefficient on the time variable in simple logistic regressions of the state variables on time for each subgroup. Notably, all subgroups are leaving C_1 (criminally active with no arrest history) over time, and all subgroups are moving into R (criminally inactive, but with recent 3-year arrest history) over time. Only men are increasing their share of C_2 (those with an arrest history who just became criminally active without arrest), over time. Black and Hispanic women are increasing in X over time

(rehabilitated or criminally inactive with no arrest history), while Black and White men are decreasing in X over time. Black and White men are increasing in A (arrested in the last year) over time.

Transitions

Table 2 in the main text shows the population "flows" between states from one wave to the next. Its entries are discussed in detail in the main text.

In the Methods Section, we analyze differences across demographic groups in each of the 16 possible transitions in Table 2 by running logistic regression models with random effects, using the following specification:

$$\log\left[\frac{\Pr(y_{i(t\to t+1)}=1)}{1-\Pr(y_{i(t\to t+1)}=1)}\right] = \beta_0 + \beta_1\,(\text{Age}-16)_{it} + \beta_2\,(\text{Age}-16)^2_{it} + \beta_3\,\text{Woman}_i$$
$$+\,\beta_4\,\text{Black}_i + \beta_5\,\text{Hispanic}_i + \beta_6\,(\text{Woman}_i * \text{Black}_i) + \beta_7\,(\text{Woman}_i * \text{Hispanic}_i) + u_i$$

where $y_{i(t\to t+1)}$ is a binary outcome indicating the transition between states in our theoretical model from survey wave t to wave $t+1$.

Long Run Equilibrium

Fig. S7 presents bar graphs of the simulated long run equilibriun by subgroup.

Figure S7: Equilibria

Results of the Sensitivity Simulation: Large Changes

Many proposed interventions involve large changes in some parameter. We investigate the sensitivity of X^* to large changes in each parameter. To investigate the possible effects of larger changes for each subpopulation, we vary each transition parameter one at a time over

a wide range that still ensures all probabilities are between zero and one. The graphs for all six subpopulations are located Figs. S8 through S19.

Figure S8: Black Males, Pre-Arrest

Figure S9: White Males, Pre-Arrest

Figure S10: Hispanic Males, Pre-Arrest

Figure S11: Black Females, Pre-Arrest

Figure S12: White Females, Pre-Arrest

Figure S13: Hispanic Females, Pre-Arrest

Figure S14: Black Males, Post-Arrest

Figure S15: White Males, Post-Arrest

Figure S16: Hispanic Males, Post-Arrest

Figure S17: Black Females, Post-Arrest

Figure S18: White Females, Post-Arrest

Figure S19: Hispanic Females, Post-Arrest

Systems Effects

We examined the result of "closing the gap." More precisely, we ran our dynamic simulation (1) with the transition parameters for White men with one exception; we changed one parameter to the corresponding value for Black men. We repeated this for each parameter. The results are pictured in Fig. S20 for the impact on X^*, and Fig. S21 for the impact on total crime $C_1^* + C_2^* + A^*$. The $X \to A$ transition dramatically constituted the largest percent gap; the impact of closing this gap dwarfs the impact of closing any of the other gaps.

Figure S20: Give White Men Black Men's Probabilities (X^*)

Figure S21: Give White Men Black Men's Probabilities (Total Crime)
